# Gold-catalyzed glycosidation for the synthesis of trisaccharides by applying the armed–disarmed strategy

**DOI:** 10.3762/bjoc.9.252

**Published:** 2013-10-18

**Authors:** Abhijeet K Kayastha, Srinivas Hotha

**Affiliations:** 1 Department of Chemistry, Indian Institute of Science Research and Education, Pune-411 008, India

**Keywords:** alkynes, armed–disarmed effect, glycosidation, gold

## Abstract

The synthesis of oligosaccharides is still a challenging task as there is no universal glycosyl donor for the synthesis of all oligosaccharides. The gold catalysis for glycosidation reactions, in which alkynylated glycosides are used, has emerged as one of the versatile options in this regard. A cleavage of the interglycosidic bond that was thought to be due to the higher reaction temperature and the acidic medium was observed during the synthesis of trisaccharides. In addition, a very little percentage of deprotection of benzyl protecting groups at the C-6 position was observed and no deprotection of benzyl ethers in aliphatic molecules was noticed. In order to overcome this fact, a collection of leaving groups that contain an alkynyl moiety were screened. It was found that 1-ethynylcyclohexanyl (Ech) glycosides are suitable for carrying out the glycosidation at 25 °C in the presence of 5 mol % each of AuCl_3_ and AgSbF_6_. Subsequently, Ech-glycosides were observed to be suitable for the synthesis of trisaccharides under gold catalysis conditions.

## Introduction

Observations that gold(III) has a great affinity for alkynes placed the chemistry of gold in an enviable situation that culminated into the total synthesis of several natural products, in which gold-mediated reactions are a key step [1–7]. Over the last two decades, chemistry with gold complexes has gained immense significance and thus been investigated for a variety of organic transformations in homogeneous and heterogeneous reaction media [8–14]. The use of gold catalysts in carbohydrate chemistry was first reported for the oxidation of alcohols [11–16]. However, until recently these catalysts were scarcely applied. Glycosidation is one of the key reactions in chemistry of carbohydrates, in which a nucleophile attaches to a saccharide to form a glycoside. In this process, the saccharide unit that is donating its glycon is called a glycosyl donor, whereas the saccharide that is accepting the glycon is referred to as glycosyl acceptor or aglycon. The synthesis of oligosaccharides is still a formidable task in spite of the development of various methods. There is still no universal glycosyl donor [17–18], although the first glycoside was reported by Emil Fischer more than a century ago.

A series of observations in our laboratory led to the identification of a gold(III)-catalyzed glycosidation reaction that uses alkynyl glycosides as glycosyl donors [19–21]. The salient features of this glycosidation reaction are the requirement of a catalytic amount of gold salts, good reaction yields and mild reaction conditions [22]. The alkynophilicity of gold(III) salts has been found to be beneficial for the synthesis of 1,2-*trans*-glycosides [23], amino acid glycoconjugates [24], carbohydrate epitopes present on the cell surface of infectious bacteria [25], glycopolypeptides [26], glycopolyacrylates [27], and glycomimetics [28]. The remarkable reactivity and chemoselectivity have also attracted other groups to investigate gold catalysts for glycosidation [29–34].

**Scheme 1:**
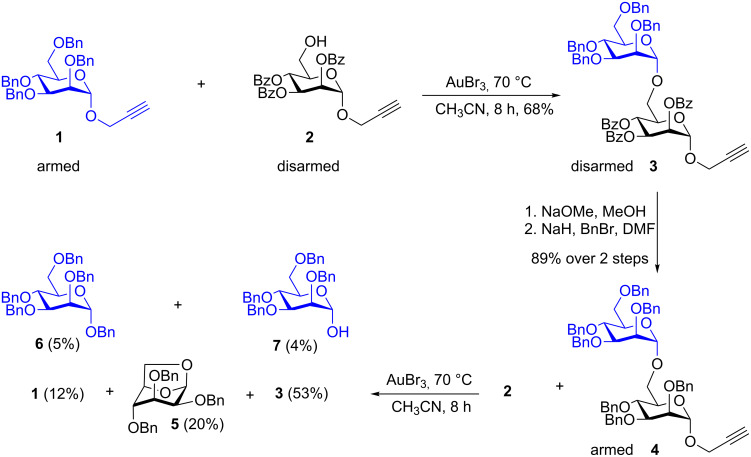
Gold-catalyzed synthesis of a disaccharide.

Esters at the C-2 position of the saccharide are known to impede the glycoside formation whereas ethers (–OBn) facilitate the reaction. Fraser-Reid applied the terms *disarmed* to deactivated glycosyl donors [e.g., esters], and *armed* to the activated donors [e.g., ethers] [35–36]. During the synthesis of oligosaccharides by sequentially adding saccharides, armed–disarmed effects can effectively be utilized to tune the reactivity of the glycosyl donors by placing appropriate protecting groups at the C-2 position. Similar armed and disarmed effects were noticed during several gold-catalyzed glycosidations [22–28]. Propargyl mannopyranosides as glycosyl donors are ideal for investigating armed–disarmed strategies for the synthesis of oligosaccharides, because the gold-catalyzed glycosidation proceeds in a highly 1,2-*trans* diastereoselective fashion [22]. Accordingly, the armed mannosyl donor **1** was allowed to react with the disarmed aglycon **2**, under the standard conditions for a gold-catalyzed glycosidation (AuBr_3_, CH_3_CN, 70 °C), to observe the formation of disaccharide **3,** in which the propargyl substitution is disarmed due to the presence of benzoates. Subsequently, the disarmed disaccharide **3** was transformed into an armed glycosyl donor **4** by simple saponification followed by etherification. The reaction between armed donor **4** and disarmed aglycon **2**, which was carried out under the aforementioned conditions did not result in the formation of desired trisaccharide. Instead, disaccharide **3** (53%) and 1,6-anhydro sugar **5** (20%) were isolated as major products [37]. Interestingly, propargyl mannoside **1** (12%) along with benzyl glycoside **6** and lactol **7** were noticed in 5% and 4% yield, respectively (Scheme 1).

**Scheme 2:**
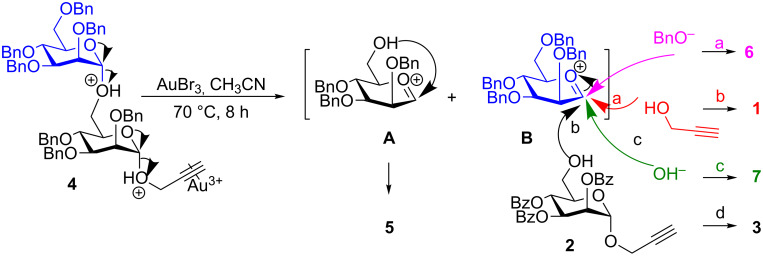
Mechanistic rationale for the cleavage of the interglycosidic linkage.

The Brønsted acid (HBr) released from AuBr_3_ in the presence of the aglycon can protonate the exocyclic oxygen present in the disaccharide **4**. The protonation of the exocyclic oxygen and subsequent cleavage could give rise to oxocarbenium ion intermediates **A** and **B** as shown in Scheme 2. The formation of 1,6-anhydro sugar **5** can be easily envisioned by the intramolecular attack of C-6-OH on the intermediate **A**. The surprising cleavage of the interglycosidic linkage leads to intermediate **B**, which can be trapped by various nucleophiles that are present in the reaction mixture. The trapping of the intermediate **B** by propargyl alcohol gives propargyl mannoside **1** (12%), the addition of OH^−^ due to moisture results in lactol **7** (4%), the addition of aglycon **2** gives rise to disaccharide **3**. The formation of benzyl mannoside **6** (5%) can be explained by the attack of BnO^−^ on the intermediate **B**. The presence of BnO^−^ could be explained due to the hydrolysis of the primary benzyl ether.

## Results and Discussion

**Scheme 3:**
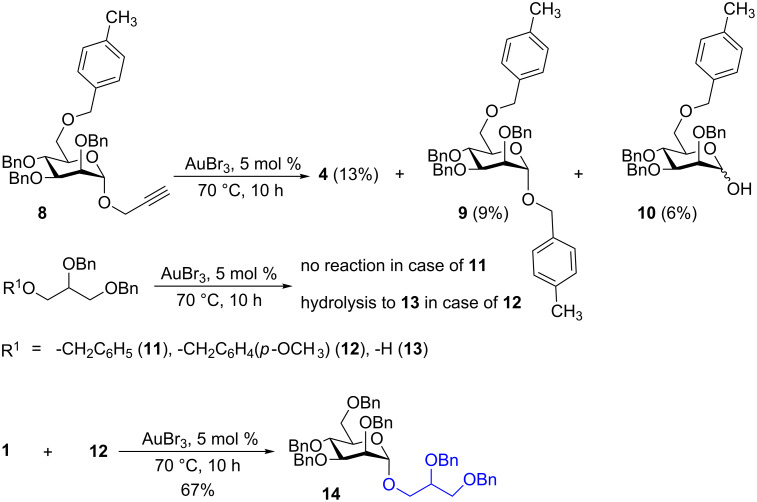
C-6 Benzyl ether hydrolysis and synthesis of a glycerol mannoside.

In order to further understand the cleavage of the C-6 benzyl ether, the model propargyl mannoside **8** was treated with 5 mol % of AuBr_3_ under aforementioned conditions. LC–MS analysis of the reaction mixture showed the formation of anhydro sugar **4** (13%), *p*-methylbenzyl mannoside **9** (9%) and lactol **10** (6%), which indicated the hydrolysis of the primary benzyl ether. The gold-catalyzed hydrolysis of benzyl ethers was not observed in the case of non-carbohydrate benzyl ether **11** (Scheme 3). For example, per-*O*-benzylated glycerol **11** did not show any benzyl deprotection, whereas the more acid-sensitive *p*-methoxybenzyl derivative **12** underwent deprotection of the *p*-methoxybenzyl moiety to give alcohol **13** with 88% yield. The deprotection of the *p*-methoxybenzyl moiety can be utilized for the one-pot synthesis of glycerol mannosides from mannosyl donor **1** and compound **12** in 67% yield under gold-catalysis conditions (Scheme 3). Importantly, the hydrolysis of benzyl ethers was not observed when the gold catalysis reactions were performed at room temperature [23–28,38].

From the above observations, the high temperature (70 °C) of the glycosidation and the oxophilicity of gold salts were observed to be major impediments for the synthesis of oligosaccharides. In order to overcome this problem, a systematic investigation of various leaving groups that bear an alkynyl moiety was carried out. The aim was to find a better leaving group, which would facilitate the glycosidation at ambient temperature. Accordingly, a panel of alkynylated glycosyl donors (**15a**–**j**) was synthesized and subjected to the glycosidation with three widely available gold salts, namely AuBr_3_, AuCl_3_ and HAuCl_4_, at 25 °C for 12 h in acetonitirile (Table 1). Substitutions at the terminal alkyne carbon (**15b**,**e**) were not tolerated. The *gem*-dimethyl alkyne **15g** showed a substantial improvement of the performance at 25 °C compared to the other alkynyl donors **15a**–**f** (Table 1). However, the *gem*-dimethyl donor **15g** was not preferred due to the shorter shelf life. The alicyclic derivatives **15h–j** gave comparable yields to **15g** and were observed to be much more stable. Furthermore, **15h** needs to be prepared from cyclohexanone, while **15i** is costly compared to **15j**. Thus, further studies were performed with **15j** only. The alkyne moiety is really essential for the transglycosylation reaction as only very little formation of the desired product was noticed in the case of the donors **15k** and **15l**. Subsequently, it was found that the addition of 5 mol % of AgSbF_6_ along with AuCl_3_ would increase the yield of disaccharide **17** to 96%. However, the disaccharide formation was not observed with AgSbF_6_ alone [38].

**Table 1:** Room temperature activation.

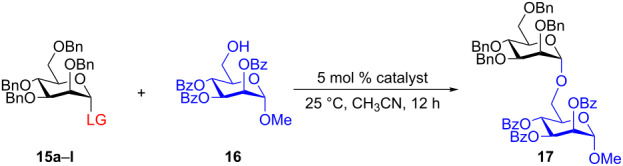							

LG	% yield of disaccharide **17** with catalyst			LG	% yield of disaccharide **17** with catalyst		
	AuCl_3_	AuBr_3_	HAuCl_4_		AuCl_3_	AuBr_3_	HAuCl_4_

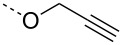 **15a**	8	15	0	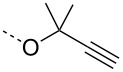 **15g**	30	8	10
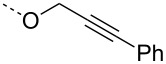 **15b**	10	15	9	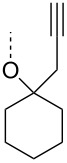 **15h**	30	13	16
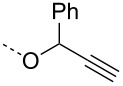 **15c**	17	0	8	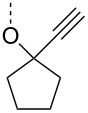 **15i**	23	8	14
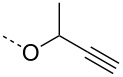 **15d**	20	5	0	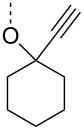 **15j**	32	16	15
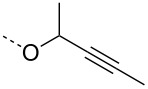 **15e**	12	0	5	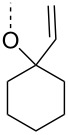 **15k**	7	3	5
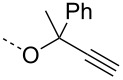 **15f**	0	0	15	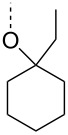 **15l**	2	0	2

**Scheme 4:**
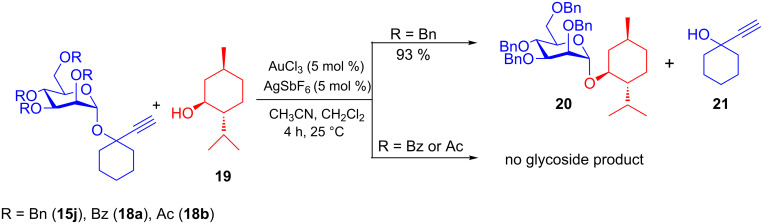
Armed–disarmed effect in Ech-glycosides during gold-catalyzed reactions.

In addition, armed mannosyl donor **15j** reacted with aglycon **19** in the presence of 5 mol % each of AuCl_3_/AgSbF_6_ in CH_3_CN/CH_2_Cl_2_ (1:1) at 25 °C for 4 h to give 1,2-*trans* menthyl mannoside **20**. The leaving group **21** could be removed easily by applying high vacuum. Disarmed donors **18a** and **18b** failed to react with menthol (**19**) under aforementioned modified gold-catalysis conditions (Scheme 4).

**Scheme 5:**
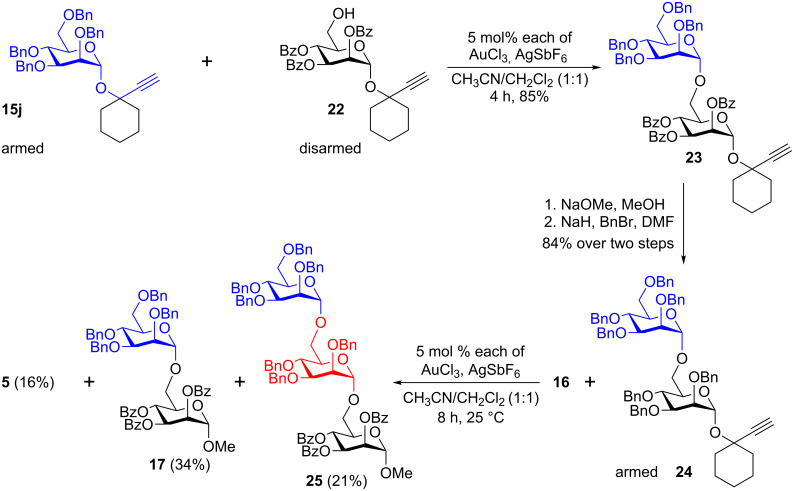
Gold-catalyzed glycosidation at ambient temperature for the synthesis of trimannoside **25**.

The strong armed–disarmed effects that were observed for the Ech-donors at 25 °C encouraged us to continue the use of the armed–disarmed strategy for the trisaccharide synthesis. Accordingly, the armed mannosyldonor **15j** was allowed to react with disarmed aglycon **22** in the presence of AuCl_3_ (5 mol %)/AgSbF_6_ (5 mol %) in CH_3_CN/CH_2_Cl_2_ (1:1) at 25 °C for 4 h to obtain the disarmed disaccharide **23** in 85% yield. Further, the armed disaccharide **24** was synthesized from **23** by saponification followed by the etherification in 84% over two steps. The glycosylation between disaccharide **24** and disarmed aglycon **16** was performed under aforementioned conditions for a gold-catalyzed transglycosidation. Purification by conventional silica gel column chromatography enabled us to characterize the anticipated trisaccharide **25** (21%) along with disaccharide **17** and anhydro sugar **5** (Scheme 5). In trisaccharide **25**, three anomeric protons were noticed at δ 4.88 (d, *J* = 1.6 Hz, 1H), 4.91 (d, *J* = 1.6 Hz, 1H), 5.61 (dd, *J* = 1.6, 3.2 Hz, 1H) ppm. The ^13^C NMR spectrum revealed that there are three mannose residues with 1,2-*trans* configuration as their anomeric carbon atoms were noticed at δ 98.1, 98.2 and 98.5 ppm and the molecular weight was found to be 1483.586 ([M + 23]^+^ for the Na adduct). The rest of the resonances in the spectrum were completely in agreement with the assigned structure of trisaccharide **25**. Formation of disaccharide **17** (34%) and anhydro sugar **5** (16%) can be rationalized on the basis of an interglycosidic bond cleavage.

**Scheme 6:**
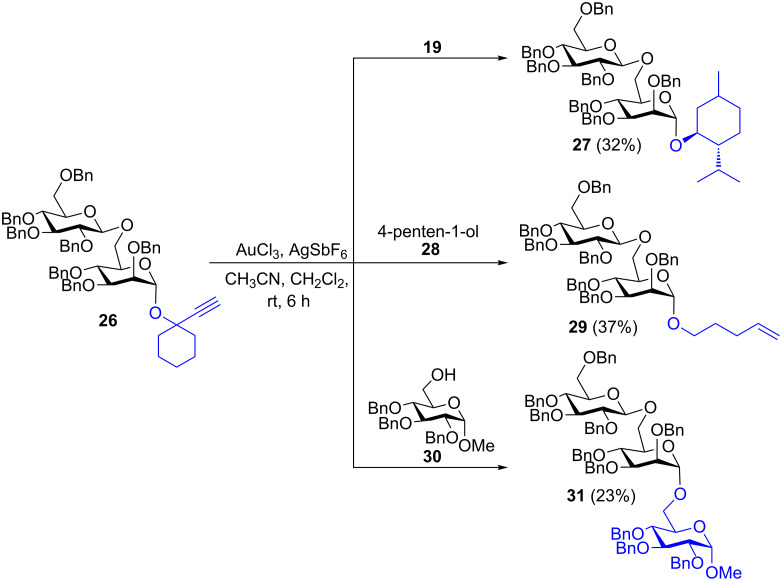
Gold-catalyzed glycosidation at ambient temperature for the synthesis of higher saccharides.

The hydrolysis of the interglycosidic bond during the gold-catalyzed transglycosidation reaction depends on the nature of interglycosidic linkage. Generally the glycosyl donors with axial hydroxy groups are considered to be more reactive than the glycosyl donors without axial hydroxy group. For example, β-D-glucose is less reactive than α-D-glucose or α-D-mannose. In order to verify the effect of the differences in reactivity on the cleavage of the interglycosidic bond, armed disaccharide **26** was prepared and allowed to react with menthol (**19**) under aforementioned conditions for 6 h to obtain the anticipated menthyl glycoside (**27**) in 32% yield. Similarly, the reactions with 4-penten-1-ol (**28**) and methyl 2,3,4-tri-*O*-benzyl α-D-glucopyranoside (**30**) gave the corresponding transglycosides **29** and **31** in 37% and 23% yield, respectively (Scheme 6).

**Scheme 7:**
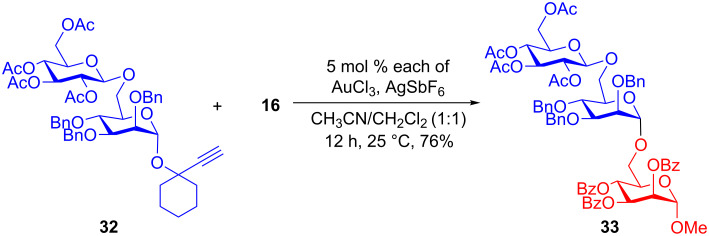
Gold-catalyzed glycosidation for synthesis of higher saccharides.

Finally, the gold-catalyzed transglycosidation reaction between disaccharide **32** and aglycon **16** gave the corresponding trisaccharide **33** in 76% yield. A cleavage of the interglycosidic bond was not observed, which shows the importance of the protecting groups in gold-catalyzed glycosidation reactions (Scheme 7).

## Conclusion

In conclusion, the armed–disarmed effect in propargyl glycosides in the presence of a catalytic amount of gold salts is studied. The high temperature of the glycosidation was found to be partially responsible for the cleavage of the interglycosidic bond along with side reactions like benzyl deprotection. These observations were then successfully applied for PMB deprotection and one-pot glycosidation. Subsequent experiments proved the significance of the alkyne moiety. It was also observed that the addition of the silver salt AgSbF_6_ during the gold-mediated transglycosidation reaction helps in reducing the reaction temperature to 25 °C. This was successfully utilized for activating 1-ethynylcyclohexanyl donors at 25 °C. Trisaccharides were synthesized under identified conditions in moderate yields.

## Experimental

All the reactions were performed under argon atmosphere. Products obtained as solids or syrups were dried under high vacuum. Gold and silver salts were purchased from Sigma-Aldrich. Analytical thin-layer chromatography was performed on pre-coated Merck silica plates (F_254_, 0.25 mm thickness); compounds were visualized by UV light or by staining with anisaldehyde spray. Optical rotations were measured on a JASCO P-1020 or Rudolph polarimeter. NMR spectra were recorded either on a Bruker AC 200, AV 400, AV 500 or JEOL ECX 400 or Bruker Avance 500 with CDCl_3_ as the solvent and tetramethylsilane as internal standard. High resolution mass spectroscopy (HRMS) was performed on ABI–MALDI–TOF using TiO_2_ as the solid matrix.

### Compound characterization data

Characterization data for compound **15j** [38]: [α]_D_^25^ +28.2 (CHCl_3_, *c* 1.00); ^1^H NMR (200.13 MHz, CDCl_3_) δ 1.10–2.15 (m, 10H), 2.40 (s, 1H), 3.65–4.12 (m, 6H), 4.59 (s, 2H), 4.60 (ABq, *J* = 12.6 Hz, 2H), 4.71 (ABq, *J* = 10.6 Hz, 2H), 4.76 (s, 2H), 5.56 (d, *J* = 1.8 Hz, 1H), 7.13–7.42 (m, 20H); ^13^C NMR (50.32 MHz, CDCl_3_) δ 22.7, 22.7, 25.0, 37.6, 38.2, 69.3, 71.9, 72.1, 72.3, 73.3, 74.1, 75.0, 75.2, 75.2, 75.5, 80.0, 84.6, 94.0, 127.3–128.3, 138.4, 138.5, 138.5, 138.5; HRMS (MALDI–TOF, *m*/*z*): [M + Na]^+^ calcd for C_42_H_46_NaO_6_, 669.3192; found, 669.3173.

Characterization data for compound **25**: [α]_D_^25^ −10.6 (CHCl_3_, *c* 1.00); ^1^H NMR (500.13 MHz, CDCl_3_) δ 3.42 (s, 3H), 3.51–3.69 (m, 8H), 3.80 (dd, *J* = 3.9, 11.6 Hz, 1H), 3.84–3.88 (m, 4H), 3.95 (dt, *J* = 9.4, 25.7 Hz, 2H), 4.14 (dt, *J* = 4.2, 9.6 Hz, 1H), 4.35–4.62 (m, 8H), 4.41 (ABq, *J* = 11.0 Hz, 2H), 4.61 (s, 2H), 4.84 (ABq, *J* = 11.0 Hz, 2H), 4.88 (d, *J* = 1.5 Hz, 1H), 4.90 (d, *J* = 1.5 Hz, 1H), 5.03 (t, *J* = 10.0 Hz, 1H), 5.06 (d, *J* = 1.3 Hz, 1H), 5.84 (dd, *J* = 3.3, 10.2 Hz, 1H), 7.11–7.51 (m, 44H), 7.81–8.08 (m, 6H); ^13^C NMR (125.76 MHz, CDCl_3_) δ 55.4, 65.6, 66.6, 69.0, 69.1, 69.8, 70.6, 71.3, 71.7, 71.7, 71.7, 71.7, 72.2, 72.7, 73.2, 74.2, 74.6, 74.8, 74.9, 74.9, 75.0, 79.2, 80.2, 98.1, 98.2, 98.4, 127.2–129.8, 133.1, 133.3, 133.5, 138.3, 138.3, 138.4, 138.4, 138.6, 138.6, 138.7, 165.3, 165.4, 165.5; HRMS (MALDI–TOF, *m*/*z*): [M + Na]^+^ calcd for C_89_H_88_NaO_19_, 1483.5818; found, 1483.5837.

Characterization data for compound **29**: [α]_D_^25^ 18.4 (CHCl_3_, *c* 1.00); ^1^H NMR (399.78 MHz, CDCl_3_) δ 1.54 (t, *J* = 7.2 Hz, 2H), 1.59 (s, 2H), 1.99 (m, 2H), 3.26–3.98 (m, 12H), 4.25–5.06 (m, 18H), 5.73 (m, 1H), 7.15–7.37 (m, 35H); ^13^C NMR (100.53 MHz, CDCl_3_) δ 28.4, 30.2, 66.8, 68.9, 69.0, 71.3, 72.0, 72.6, 73.4, 74.7, 74.7, 74.9, 74.9, 74.9, 75.0, 75.7, 77.8, 80.2, 82.0, 84.6, 97.7, 104.0, 114.8, 126.9–128.5, 137.9, 138.1, 138.2, 138.3, 138.5, 138.5, 138.6; HRMS (MALDI–TOF, *m*/*z*): [M + Na]^+^ calcd for C_66_H_72_NaO_11_, 1063.4972; found, 1063.4994.

Characterization data for compound **31**: [α]_D_^25^ +23.1 (CHCl_3_, *c* 1.00); ^1^H NMR (399.78 MHz, CDCl_3_) δ 3.17 (s, 3H), 3.25–3.94 (m, 17H), 4.11 (dd, *J* = 1.8, 9.1 Hz, 1H), 4.24–4.96 (m, 23H), 7.04–7.32 (m, 50H); ^13^C NMR (100.53 MHz, CDCl_3_) δ 55.0, 65.6, 68.7, 69.0, 69.8, 71.3, 71.8, 72.5, 73.2, 73.4, 73.4, 74.5, 74.6, 74.7, 74.8, 74.9, 74.9, 74.9, 74.9, 77.5, 77.8,79.5, 79.9, 82.0, 82.0, 84.6, 97.6, 98.1, 104.0, 127.3–128.4, 138.1, 138.1, 138.1, 138.2, 138.3, 138.3, 138.5, 138.6, 138.6, 138.7; HRMS (MALDI–TOF, *m*/*z*): [M + Na]^+^ calcd for C_89_H_94_NaO_16_, 1441.6440; found, 1441.6457.

Characterization data for compound **33**: [α]_D_^25^ −63.8 (CHCl_3_, *c* 1.00); ^1^H NMR (399.78 MHz, CDCl_3_) δ 1.95 (s, 3H), 1.99 (s, 3H), 2.01 (s, 3H), 2.02 (s, 3H), 3.50–4.21 (m, 13H), 4.33–4.37 (m, 3H), 4.52 (d, *J* = 11.4 Hz, 1H), 4.58 (s, 2H), 4.90–4.96 (m, 3H), 5.01 (t, *J* = 9.8 Hz, 1H), 5.03 (t, *J* = 10.1 Hz, 1H), 5.13 (t, *J* = 9.4 Hz, 1H), 5.63 (dd, *J* = 1.8, 3.2 Hz, 1H), 5.84 (dd, *J* = 3.2, 9.8 Hz, 1H), 7.23–7.54 (m, 24H), 7.81–8.11 (m, 6H); ^13^C NMR (100.53 MHz, CDCl_3_) δ 20.5, 20.5, 20.6, 20.6, 55.4, 61.8, 66.6, 67.8, 68.3, 68.7, 69.1, 69.9, 70.5, 71.0, 71.1, 71.5, 71.5, 72.5, 72.8, 74.4, 74.6, 74.8, 80.1, 98.0, 98.4, 100.9, 127.4–129.8, 133.0, 133.3, 133.4, 138.2, 138.3, 138.5, 165.3, 165.4, 165.5, 169.0, 169.4, 170.3, 170.6; HRMS (MALDI–TOF, *m*/*z*): [M + Na]^+^ calcd for C_69_H_72_NaO_23_, 1291.4362; found, 1291.4377.

## Supporting Information

File 1 Detailed experimental data.
