# Supplementary material for: Gold-catalyzed glycosidation for the synthesis of trisaccharides by applying the armed–disarmed strategy
Source: Beilstein J Org Chem. 2013 Oct 18;9:2147–55. doi: 10.3762/bjoc.9.252 (PMC3817485; doi:10.3762/bjoc.9.252)
Supplement: File 1 — Detailed experimental data. [file Beilstein_J_Org_Chem-09-2147-s001.pdf]

Supporting Information

for

Gold-catalyzed glycosidation for the synthesis of trisaccharides by applying the armed–disarmed strategy

Abhijeet K. Kayastha and Srinivas Hotha*

Address: Department of Chemistry, Indian Institute of Science Research and Education, Pune-411 008, India

Email: Srinivas Hotha* - s.hotha@iiserpune.ac.in

* Corresponding author

Detailed experimental data

Contents

	Page No.
General experimental procedures	S2
Compound characterization data	S3–S10
^{1}H, ^{13}C and DEPT NMR spectral charts of compounds **6**, **7**, **8**, **12**, **13**, **14, 15j, 15k**, **15l**, **17**, **24**, **25**, **26**, **27**, **29**, **31**, **32**, **33**	S11–S28
References	S29

General experimental procedure

To a solution of glycosyl donor (1 equiv) and aglycon (1.1 equiv) in 3 mL CH_3CN/CH_2Cl_2 (1:1) was added a solution of $AuCl_3$ (5 mol %) and $AgSbF_6$ (5 mol %) in 3 mL of CH_3CN/CH_2Cl_2 (1:1) and the mixture was stirred at 25 °C for 4 h under argon atmosphere. After completion of the reaction (judged by TLC), the dark brown reaction mixture was concentrated in vacuo and the crude residue was purified through silica gel column chromatography using ethyl acetate/petroleum ether (1:5) to give the corresponding transglycosides.

Compound characterization data

Characterization data for compound **3** [1]:

$[\alpha]_D^{25}$ = -23.3 ($CHCl_3$, *c* 1.0); ^{1}H NMR (200.13 MHz, $CDCl_3$): δ 2.50 (t, 1H, *J* = 2.4 Hz), 3.53–3.78 (m, 4H), 3.84 (dd, 1H, *J* = 3.3, 9.2 Hz), 3.88–4.05 (m, 2H), 4.22 (m, 1H), 4.31 (d, 2H, *J* = 2.4 Hz), 4.37 (d, 2H, *J* = 4.5 Hz), 4.43 (s, 2H) 4.49 (ABq, 2H, *J* = 12.3 Hz), 4.63 (s, 2H), 4.86 (d, 1H, *J* = 10.9 Hz), 4.96 (d, 1H, *J* = 1.8 Hz), 5.26 (d, 1H, *J* = 1.8 Hz), 5.68 (dd, 1H, *J* = 1.8, 2.9 Hz), 5.89 (m, 2H),7.10–7.56 (m, 29H), 7.75–8.11 (m, 6H); ^{13}C NMR (50.32 MHz, $CDCl_3$): δ 55.1, 66.6, 67.1, 69.0, 69.7, 69.8, 70.4, 71.8, 71.9, 72.5, 73.2, 74.7, 74.8, 74.9, 75.7, 78.1, 80.1, 96.2, 98.2, 127.3–128.9, 133.1, 133.3, 133.5, 138.3, 138.4, 138.5, 138.6,165.3, 165.4, 165.4; HRMS (MALDI-TOF): calcd. for $C_{64}H_{60}NaO_{14}$[M^+ +Na]: 1075.388; found: 1075.3889.

Characterization data for compound **4** [1]

$[\alpha]_D^{25}$ = +31.4 ($CHCl_3$, *c* 1.0); ^{1}H NMR (200.13 MHz, $CDCl_3$): δ 2.37 (t, 1H, *J* = 2.4 Hz), 3.58–4.07 (m, 12H), 4.10 (dd, 2H, *J* = 0.7, 2.4 Hz), 4.42–4.71 (m, 8H), 4.61 (s, 2H), 4.68 (s, 2H), 4.87 (d, 2H, *J* = 10.8 Hz), 4.99 (d, 1H, *J* = 1.6 Hz), 5.11 (d, 1H, *J* = 1.6 Hz), 7.10–7.45 (m, 35H); ^{13}C NMR (100.61 MHz, $CDCl_3$): δ 54.1, 65.9, 69.1, 71.5, 71.8, 72.0, 72.1, 72.4, 72.9, 73.2, 74.4, 74.6, 74.7, 74.7, 74.9, 74.9, 75.0, 78.8, 79.3, 80.0, 96.5, 98.1, 127.3–128.4, 138.1, 138.4,138.4,138.4,138.4, 138.6, 138.7; HRMS (MALDI-TOF): calcd. for $C_{64}H_{66}NaO_{11}$[M^+ +Na]: 1033.4503; found: 1033.4510.

Characterization data for compound **5** [1]

$[\alpha]_D^{25}$ = -16.6 ($CHCl_3$, *c* 1.0); ^{1}H NMR (200.13 MHz, $CDCl_3$): δ 3.47 (t, 1H, *J* = 1.8 Hz), 3.58 (dd, 1H, *J* = 1.8, 5.4 Hz), 3.73 (dd, 1H, *J* = 6.0, 7.1 Hz), 3.81 (qd, 1H, *J* = 1.6, 3.1, 5.0 Hz), 4.25 (dd, 1H, *J* = 0.9, 7.1 Hz), 4.43–4.57 (m, 5H), 4.52 (ABq, 2H, *J* = 12.4 Hz), 5.46 (s,

1H), 7.20–7.38 (m, 15H); ^{13}C NMR (50.32 MHz, $CDCl_3$): δ 65.0, 71.3, 71.4, 73.4, 74.1, 74.4, 74.5, 76.5, 100.1, 127.7–128.5, 137.6, 137.9, 137.9; Mol. Wt. Calculated for $C_{27}H_{28}NaO_5$[M$^+$ +Na]: 455.1825; found: 455.1830.

Characterization data for compound **6**

$[\alpha]_D^{25}$ = -38.5 ($CHCl_3$, *c* 1.0);^{1}H NMR (200.13 MHz, $CDCl_3$): δ 3.45–3.51 (m, 2H), 3.79–3.96 (m, 4H), 4.67 (ABq, 2H, *J* = 3.0 Hz), 4.69 (ABq, 2H, *J* = 5.2 Hz), 4.56-4.70 (m, 4H), 4.92 (m, 2H), 5.05 (m, 1H), 7.17-7.47 (m, 25H); ^{13}C NMR (50.32 MHz, $CDCl_3$): 69.6, 70.7, 71.4, 73.4, 73.8, 73.8, 74.8, 75.0, 75.9, 82.3, 100.2, 127.2-128.3, 137.4, 138.0, 138.3, 138.4, 138.7; HRMS (MALDI-TOF): calcd. for $C_{41}H_{42}NaO_6$[M$^+$ +Na]: 653.2879; found: 653.2875.

Characterization data for compound **7**

$[\alpha]_D^{25}$ = +9.5 ($CHCl_3$, *c* 1.0);^{1}H NMR (200.13 MHz, $CDCl_3$): δ 3.42–4.08 (m, 7H), 4.53 (m, 1H), 4.60 (s, 2H), 4.72 (d, 2H, *J* = 1.9 Hz), 4.72 (ABq, 2H, *J* = 11.3 Hz), 4.85 (t, 1H, *J* = 5.5 Hz), 5.24 (d, 1H, *J* = 1.2 Hz); ^{13}C NMR (50.32 MHz, $CDCl_3$): 69.6, 71.5, 72.1, 72.6, 73.2, 74.8, 75.0, 75.2, 79.7, 92.7, 127.6-128.5, 138.0, 138.1, 138.3, 138.5; HRMS (MALDI-TOF): calcd. for $C_{34}H_{36}NaO_6$[M$^+$ +Na]: 563.2410; found: 563.2416.

Characterization data for compound **8**

$[\alpha]_D^{25}$= +34.7 ($CHCl_3$, *c* 1.0);^{1}H NMR (399.78 MHz, $CDCl_3$): δ 2.31 (s, 3H), 2.38 (t, 1H, *J* = 2.4 Hz), 3.68-3.77 (m, 3H), 3.83 (dd, 1H, *J* = 1.9, 1.2 Hz), 3.88 (dd, 1H, *J* = 3.2, 9.2 Hz), 3.99 (t, 1H, *J* = 9.5 Hz), 4.10 (d, 2H, *J* = 2.2 Hz), 4.58 (ABq, 2H, *J* = 11.9 Hz), 4.59 (s, 2H), 4.65 (ABq, 2H, *J* = 11.0 Hz), 4.73 (q, 2H, *J* = 2.8, 15.2 Hz), 5.08 (d, 1H, *J* = 1.8 Hz), 7.09-7.39 (m, 19H); ^{13}C NMR (100.53 MHz, $CDCl_3$): 21.2, 54.0, 68.7, 72.0, 72.2, 72.6, 73.2, 74.3, 74.6, 74.7, 75.0, 78.8, 79.9, 96.4, 127.4-128.9, 135.1, 137.1, 138.1, 138.3, 138.4; Mol. Wt. Calculated for $C_{38}H_{40}NaO_6$ [M$^+$ +Na]: 615.2723; found: 615.2731.

Characterization data for compound **12**

$[\alpha]_D^{25}$= N/R (racemic);^{1}H NMR (399.78 MHz, $CDCl_3$): δ 3.56–3.65 (m, 4H), 3.80 (s, 4H), 4.46 (s, 2H), 4.53 (s, 2H), 4.69 (s, 2H), 6.86 (d, 2H *J* = 8.2 Hz), 7.22-7.34 (m, 12H); ^{13}C NMR (100.53 MHz, $CDCl_3$): 55.2, 69.9, 70.3, 72.1, 73.0, 73.3, 77.2, 113.7, 127.4-130.3, 138.2, 138.6, 159.1; HRMS (MALDI-TOF): calcd. for $C_{25}H_{28}NaO_4$[M^+ +Na]: 415.1885; found: 415.1889.

Characterization data for compound **13**

$[\alpha]_D^{25}$= N/R (racemic);^{1}H NMR (200.13 MHz, $CDCl_3$): δ 2.15 (bs, 1H), 3.59-3.73 (m, 5H), 4.54 (s, 2H), 4.65 (dd, 2H, *J* = 10.1, 12.0 Hz), 7.24-7.36 (m, 10H); ^{13}C NMR (50.32 MHz, $CDCl_3$): 62.8, 70.1, 72.1, 73.5, 78.0, 127.6-128.4, 137.9, 138.2; HRMS (MALDI-TOF): calcd. for $C_{17}H_{20}NaO_3$[M^+ Na]: 295.1310; found: 295.1318.

Characterization data for compound **14**

$[\alpha]_D^{25}$ = N/R (racemic);^{1}H NMR (399.78 MHz, $CDCl_3$): δ 3.50-3.57 (m, 3H), 3.65-3.87 (m, 7H), 4.00 (m, 1H), 4.46-4.52 (m, 4H), 4.59-4.70 (m,7H), 4.86-4.90 (m, 2H), 7.15-7.36 (m, 30H); ^{13}C NMR (100.53 MHz, $CDCl_3$): 67.2, 69.0, 69.8, 71.7, 72.1, 72.2, 72.4, 73.2, 73.3, 74.6, 74.8, 75.0, 76.7, 79.9, 98.0, 127.4-128.3, 138.1, 138.3, 138.3, 138.3, 138.5, 138.6; HRMS (MALDI-TOF): calcd. for $C_{51}H_{54}NaO_8$ [M^+ +Na]: 817.3716; found: 817.3723.

Characterization data for compound **15j** [2]

$[\alpha]_D^{25}$ = +28.2 ($CHCl_3$, *c* 1.00); ^{1}H NMR (200.13 MHz, $CDCl_3$): δ 1.10-2.15 (m, 10H), 2.40 (s, 1H), 3.65-4.12 (m, 6H), 4.59 (s, 2H), 4.60 (ABq, 2H, *J* = 12.6 Hz), 4.71 (ABq, 2H, *J* = 10.6 Hz), 4.76 (s, 2H), 5.56 (d, 1H, *J* = 1.8 Hz), 7.13-7.42 (m, 20H); ^{13}C NMR (50.32 MHz, $CDCl_3$):δ 22.7, 22.7, 25.0, 37.6, 38.2, 69.3, 71.9, 72.1, 72.3, 73.3, 74.1, 75.0, 75.2, 75.2, 75.5,

80.0, 84.6, 94.0, 127.3-128.3, 138.4, 138.5, 138.5, 138.5; HRMS (MALDI-TOF): calcd. for $C_{42}H_{46}NaO_6$ [M^++Na]: 669.3192; found: 669.3173.

Characterization data for compound **15k**

$[\alpha]_D^{25}$ = -11.9 ($CHCl_3$, *c* 1.00); ^{1}H NMR (200.13 MHz, $CDCl_3$): δ 1.07-1.90 (m, 10H), 3.50 (t, 1H, *J* = 2.0 Hz), 3.68 (dd, 1H, *J* = 0.8, 10.3 Hz), 3.82 (dd, 1H, *J* = 3.7, 10.3 Hz), 3.92 (m, 3H), 4.59 (ABq, 2H, *J* = 12.1 Hz), 4.63 (ABq, 2H, *J* = 11.8 Hz), 4.68 (s, 2H), 4.71 (ABq, 2H, *J* = 10.6 Hz), 4.97 (d, 1H, *J* = 1.8 Hz), 5.03 (dd, 1H, *J* = 1.2, 7.2 Hz), 5.01 (s, 1H), 5.65 (m, 1H), 7.15-7.37 (m, 20H); ^{13}C NMR (50.32 MHz, $CDCl_3$):δ 21.9, 21.9, 25.5, 33.9, 35.4, 69.3, 72.0, 72.1, 72.2, 73.3, 75.1, 75.2, 75.6, 77.9, 79.8, 92.6, 115.1, 127.3-128.3, 138.3, 138.5, 138.5, 138.5, 142.7; HRMS (MALDI-TOF): calcd. for $C_{42}H_{48}NaO_6$ [M^++Na]: 671.3349; found: 671.3360.

Characterization data for compound **15l**

$[\alpha]_D^{25}$ = +48.2 ($CHCl_3$, *c* 1.00); ^{1}H NMR (200.13 MHz, $CDCl_3$): δ 0.68 (t, 3H, *J* = 7.9 Hz), 1.08-1.65 (m, 12H), 3.53 (t, 1H, *J* = 2.2 Hz), 3.68 (dd, 1H, *J* = 1.2, 9.5 Hz), 3.83 (dd, 1H, *J* = 3.3, 10.4 Hz), 3.98 (m, 3H), 4.60 (ABq, 2H, *J* = 12.0 Hz), 4.68 (ABq, 2H, *J* = 11.8 Hz), 4.68 (ABq, 2H, *J* = 10.3 Hz), 4.72 (ABq, 2H, *J* = 12.1 Hz), 4.98 (d, 1H, *J* = 1.9 Hz), 7.20-7.39 (m, 20H);^{13}C NMR (50.32 MHz, $CDCl_3$):δ 7.5, 21.9, 22.1, 25.7, 30.3, 33.6, 34.4, 69.3, 71.8, 72.4, 72.4, 73.3, 75.1, 75.2, 75.9, 78.4, 79.8, 91.4, 127.3-128.3, 138.4, 138.5, 138.6, 138.6; HRMS (MALDI-TOF): calcd. for $C_{42}H_{50}NaO_6$ [M^++Na]: 673.3505; found: 673.3507.

Characterization data for compound **17** [2]

$[\alpha]_D^{25}$ ($CHCl_3$, *c* 1.0) = -43.8; ^{1}H NMR (200.13 MHz, $CDCl_3$):δ 3.43 (s, 3H), 3.54–3.75 (m, 5H), 3.81-4.01(m 3H), 4.18 (m, 1H), 4.37 (d, 2H, *J* = 2.5 Hz), 4.49 (t, 2H, *J* = 10.3 Hz), 4.49 (ABq, 2H, *J* = 12.3 Hz), 4.68 (s, 2H), 4.95 (dd, 2H, *J* = 1.7, 5.6 Hz), 5.62 (dd, 1H, *J* = 1.8, 2.9

Hz), 5.89 (m, 2H),7.13–7.53 (m, 29H), 7.78–8.10 (m, 6H); ^{13}C NMR (125.76 MHz, $CDCl_3$): δ 55.4,66.7, 67.8, 68.9, 69.0, 69.9, 70.6, 71.7, 71.8, 72.4, 73.2, 74.6, 74.7, 75.0, 80.1, 98.1, 98.4, 127.3–129.8, 133.1, 133.3, 133.5, 138.3, 138.4, 138.5, 138.6,165.3, 165.4, 165.4; HRMS (MALDI-TOF): calcd. for $C_{28}H_{26}NaO_9$[M^++Na]: $C_{62}H_{60}NaO_{14}$, 1051.3881; found, 1051.3889.

Characterization data for compound **20** [2]

$[\alpha]_D^{25}$ = +5.2 ($CHCl_3$, *c* 1.00); ^{1}H NMR (200.13 MHz, $CDCl_3$): δ 0.64 (d, 3H, *J* = 6.7 Hz), 0.80 (d, 3H, *J* = 1.3 Hz), 0.84 (d, 3H, *J* = 2.0 Hz), 0.87-1.85 (m, 9H), 2.15 (m, 1H), 3.23 (dt, 1H, *J* = 4.3, 10.5 Hz), 3.67 (q, 1H, *J* = 2.0, 4.1 Hz), 3.76 (dd, 1H, *J* = 1.6, 4.7 Hz), 3.84-3.98 (m, 3H), 4.63 (ABq, 2H, *J* = 12.1 Hz), 4.63 (d, 1H, *J* = 1.4 Hz), 4.67 (ABq, 2H, *J* = 11.5 Hz), 4.68 (ABq, 2H, *J* = 12.1 Hz), 4.70 (ABq, 2H, *J* = 10.7 Hz), 7.10-7.38 (m, 20H);^{13}C NMR (50.32 MHz, $CDCl_3$):δ 16.2, 21.0, 22.2, 23.2, 25.7, 31.6, 34.2, 42.8, 48.6, 69.4, 71.7, 72.2, 72.4, 73.3, 74.3, 75.2, 75.2, 80.0, 81.0, 99.8, 127.3-128.4, 138.2, 138.4, 138.5, 138.5; HRMS (MALDI-TOF): calcd. for $C_{44}H_{54}NaO_6$ [M^++Na]: 701.3818; found: 701.3821.

Characterization data for compound **24**

$[\alpha]_D^{25}$ = +26.5 ($CHCl_3$, *c* 1.00); ^{1}H NMR (200.13 MHz): δ 1.26-1.73 (m, 10H), 2.41 (s, 1H), 3.61-4.01 (m, 12H), 4.43-4.69 (m, 12H), 4.87 (dd, 2H, *J* = 3.1, 10.8 Hz), 5.11 (d, 1H, *J* = 1.3 Hz), 5.46 (d, 1H, J = 1.8 Hz), 7.11-7.41 (m, 35H); ^{13}C NMR (50.32 MHz, $CDCl_3$): δ 22.6, 22.6, 25.0, 37.6, 38.1, 66.3, 69.0, 71.4, 71.6, 71.9, 72.1, 72.3, 72.3 73.1, 74.2, 74.5, 74.6, 74.8, 74.9, 75.0, 75.3, 75.3, 79.4, 79.9, 84.4, 93.9, 98.1, 127.2-128.4, 138.2, 138.3, 138.3, 138.4, 138.4, 138.4, 138.6; HRMS (MALDI-TOF): calcd. for $C_{69}H_{74}NaO_{11}$ [M^++Na]: 1101.5129; found: 1101.5134.

Characterizationdataforcompound**25**

$[\alpha]_D^{25}$ = -10.6 ($CHCl_3$, *c* 1.00); ^{1}H NMR (500.13 MHz, $CDCl_3$):δ 3.42 (s, 3H), 3.51-3.69 (m, 8H), 3.80 (dd, 1H, *J* = 3.9, 11.6 Hz), 3.84-3.88 (m, 4H), 3.95 (dt, 2H, *J* = 9.4, 25.7 Hz), 4.14 (dt, 1H, *J* = 4.2, 9.6 Hz), 4.35-4.4.62 (m, 8H), 4.41 (ABq, 2H, *J* = 11.0 Hz), 4.61 (s, 2H), 4.84 (ABq, 2H, *J* = 11.0 Hz), 4.88 (d, 1H, *J* = 1.5 Hz), 4.90 (d, 1H, *J* = 1.5 Hz), 5.03 (t, 1H, *J* = 10.0 Hz), 5.06 (d, 1H, *J* = 1.3 Hz), 5.84 (dd, 1H, *J* = 3.3, 10.2 Hz), 7.11-7.51 (m, 44H), 7.81-8.08 (m, 6H); ^{13}C NMR (125.76 MHz, $CDCl_3$): δ 55.4, 65.6, 66.6, 69.0, 69.1, 69.8, 70.6, 71.3, 71.7, 71.7, 71.7, 71.7, 72.2, 72.7, 73.2, 74.2, 74.6, 74.8, 74.9, 74.9, 75.0, 79.2, 80.2, 98.1, 98.2, 98.4, 127.2-129.8, 133.1, 133.3, 133.5, 138.3, 138.3, 138.4, 138.4, 138.6, 138.6, 138.7, 165.3, 165.4, 165.5; HRMS (MALDI-TOF): calcd. for $C_{89}H_{88}NaO_{19}$ [M^++Na]: 1483.5818; found: 1483.5837.

Characterization data for compound **26**

$[\alpha]_D^{25}$ = +20.3 ($CHCl_3$, *c* 1.00); ^{1}H NMR (399.78 MHz, $CDCl_3$): δ 1.42-1.75 (m, 10H), 2.34 (s, 1H), 3.43 (ddd, 1H, *J* = 1.8, 4.3, 9.0 Hz), 3.49-3.76 (m, 7H), 3.97 (m, 2H), 4.08 (t, 1H, *J* = 9.6 Hz), 4.23 (dd, 1H, *J* = 1.4, 3.5, Hz), 4.48-4.83 (m, 8H), 4.54 (ABq, 2H, *J* = 7.8 Hz), 4.58 (s, 2H), 4.80 (d, 1H, *J* = 2.9 Hz), 5.02 (ABq, 2H, *J* = 11.1 Hz), 5.57 (d, 1H, *J* = 1.7 Hz), 7.16-7.40 (m, 35H); ^{13}C NMR (100.53 MHz, $CDCl_3$): δ 22.7, 22.7, 24.9, 37.5, 38.2, 68.8, 69.0, 71.7, 71.9, 72.2, 73.3, 74.1, 74.8, 74.8, 74.9, 74.9, 75.0, 75.2, 75.5, 75.6, 77.9, 79.8, 82.1, 84.5, 84.7, 94.0, 104.0, 127.3-129.5, 138.1. 138.2, 138.4, 138.5, 138.6, 138.6, 138.7; HRMS (MALDI-TOF): calcd. for $C_{69}H_{74}NaO_{11}$ [M^++Na]: 1101.5129; found: 1101.5138.

Characterization data for compound **27**

$[\alpha]_D^{25}$ = +19.3 ($CHCl_3$, *c* 1.00); ^{1}H NMR (399.78 MHz, $CDCl_3$): δ 0.58 (d, 2H *J* = 6.7 Hz), 0.73 (m, 2H), 0.81 (t, 6H, *J* = 7.4Hz), 0.93 (t, 1H, *J* = 12.0 Hz), 1.06 (t, 1H, *J* = 10.7 Hz), 1.23 (t, 1H, *J* = 7.0 Hz), 1.51 (m, 2H), 1.59 (s, 2H), 1.73 (m, 1H), 2.08 (d, 1H, *J* = 12.4 Hz),

3.16 (dt, 1H, *J* = 4.4, 14.8, Hz), 3.42-4.04 (m, 11H), 4.27-5.11 (m, 11H), 4.64 (ABq, 2H, *J* = 11.1 Hz), 4.87 (d, 1H, *J* = 1.4 Hz), 5.02 (ABq, 2H, *J* = 10.8 Hz), 7.15-7.42 (m, 35H); ^{13}C NMR (100.53 MHz, $CDCl_3$): δ 16.1, 21.0, 22.2, 23.0, 25.5, 31.5, 34.1, 42.8, 48.7, 69.0, 69.2, 71.1, 72.0, 72.4, 73.4, 74.1, 74.9, 74.9, 75.0, 75.0, 75.1, 75.7, 77.8, 80.1, 80.6, 82.2, 84.6, 99.8, 104.2, 127.4-128.5, 138.1, 138.1, 138.2, 138.5, 138.6, 138.6, 138.6; HRMS (MALDI-TOF): calcd. for $C_{71}H_{82}NaO_{11}$ [M^++Na]: 1133.5755; found: 1133.5763.

Characterizationdataforcompound**29**

$[\alpha]_D^{25}$ = +18.4 ($CHCl_3$, *c* 1.00); ^{1}H NMR (399.78 MHz, $CDCl_3$): δ 1.54 (t, 2H, *J* = 7.2 Hz), 1.59 (s, 2H), 1.99 (m, 2H), 3.26-3.98 (m, 12H), 4.25-5.06 (m, 18H), 5.73 (m, 1H), 7.15-7.37 (m, 35H); ^{13}C NMR (100.53 MHz, $CDCl_3$): δ 28.4, 30.2, 66.8, 68.9, 69.0, 71.3, 72.0, 72.6, 73.4, 74.7, 74.7, 74.9, 74.9, 74.9, 75.0, 75.7, 77.8, 80.2, 82.0, 84.6, 97.7, 104.0, 114.8, 126.9-128.5, 137.9, 138.1, 138.2, 138.3, 138.5, 138.5, 138.6 ; HRMS (MALDI-TOF): calcd. for $C_{66}H_{72}NaO_{11}$ [M^++Na]: 1063.4972; found: 1063.4994.

Characterization data for compound **31**

$[\alpha]_D^{25}$ = +23.1 ($CHCl_3$, *c* 1.00); ^{1}H NMR (399.78 MHz, $CDCl_3$): δ 3.17 (s, 3H), 3.25-3.94 (m, 17H), 4.11 (dd, 1H, *J* = 1.8, 9.1 Hz), 4.24-4.96 (m, 23H), 7.04-7.32 (m, 50H); ^{13}C NMR (100.53 MHz, $CDCl_3$): δ 55.0, 65.6, 68.7, 69.0, 69.8, 71.3, 71.8, 72.5, 73.2, 73.4, 73.4, 74.5, 74.6, 74.7, 74.8, 74.9, 74.9, 74.9, 74.9, 77.5, 77.8,79.5, 79.9, 82.0, 82.0, 84.6, 97.6, 98.1, 104.0, 127.3-128.4, 138.1, 138.1, 138.1, 138.2, 138.3, 138.3, 138.5, 138.6, 138.6, 138.7; HRMS (MALDI-TOF): calcd. for $C_{89}H_{94}NaO_{16}$ [M^++Na]: 1441.6440; found: 1441.6457.

Characterization data for compound **32**

$[\alpha]_D^{25}$ = +11.5 ($CHCl_3$, *c* 1.00); ^{1}H NMR (399.78 MHz, $CDCl_3$): δ 1.25-1.72 (m, 10H), 1.99-2.03 (4s, 12H), 2.42 (s, 1H), 3.58 (dq, 1H, *J* = 2.5, 9.6 Hz), 3.71 (m, 2H), 3.80 (m, 1H), 3.92

(dd, 2H, *J* = 2.7, 9.1 Hz), 4.12 (m, 2H), 4.22 (dd, 1H, *J* = 4.6, 12.6 Hz), 4.53 (ABq, 2H, *J* = 7.8 Hz), 4.57 (s, 2H), 4.71 (ABq, 2H, *J* = 12.8 Hz), 4.95-5.16 (m, 4H), 5.48 (d, 1H, *J* = 1.6 Hz), 7.26-7.38 (m, 15H); ^{13}C NMR (100.53 MHz, $CDCl_3$): δ 20.5, 20.5, 20.6, 20.6, 22.6, 22.7, 25.0, 37.5, 38.2, 61.9, 68.3, 69.0, 71.1, 71.6, 71.7, 72.0, 72.2, 73.0, 74.3, 74.7, 74.9, 75.0, 75.7, 79.7, 84.4, 93.8, 100.7, 125.5-128.4, 138.2, 138.3, 138.4, 169.1, 169.3, 170.3, 170.7; HRMS (MALDI-TOF): calcd. for $C_{49}H_{58}NaO_{15}$ [M^++Na]: 909.3673; found: 909.3678.

Characterization data for compound **33**

$[\alpha]_D^{25}$ = -63.8 ($CHCl_3$, *c* 1.00); ^{1}H NMR (399.78 MHz, $CDCl_3$): δ 1.95 (s, 3H), 1.99 (s, 3H), 2.01 (s, 3H), 2.02 (s, 3H), 3.50-4.21 (m, 13H), 4.33-4.37 (m, 3H), 4.52 (d, 1H, *J* = 11.4 Hz), 4.58 (s, 2H), 4.90-4.96 (m, 3H), 5.01 (t, 1H, *J* = 9.8 Hz), 5.03 (t, 1H, *J* = 10.1Hz), 5.13 (t, 1H, *J* = 9.4 Hz), 5.63 (dd, 1H, *J* = 1.8, 3.2 Hz), 5.84 (dd, 1H, *J* = 3.2, 9.8 Hz), 7.23-7.54 (m, 24H), 7.81-8.11 (m, 6H); ^{13}C NMR (100.53 MHz, $CDCl_3$): δ 20.5, 20.5, 20.6, 20.6, 55.4, 61.8, 66.6, 67.8, 68.3, 68.7, 69.1, 69.9, 70.5, 71.0, 71.1, 71.5, 71.5, 72.5, 72.8, 74.4, 74.6, 74.8, 80.1, 98.0, 98.4, 100.9, 127.4-129.8, 133.0, 133.3, 133.4, 138.2, 138.3, 138.5, 165.3, 165.4, 165.5, 169.0, 169.4, 170.3, 170.6 ; HRMS (MALDI-TOF): calcd. for $C_{69}H_{72}NaO_{23}$ [M^++Na]: 1291.4362; found: 1291.4377.

^{1}H NMR Spectrum (200.13 MHz, $CDCl_3$) of Compound **6**

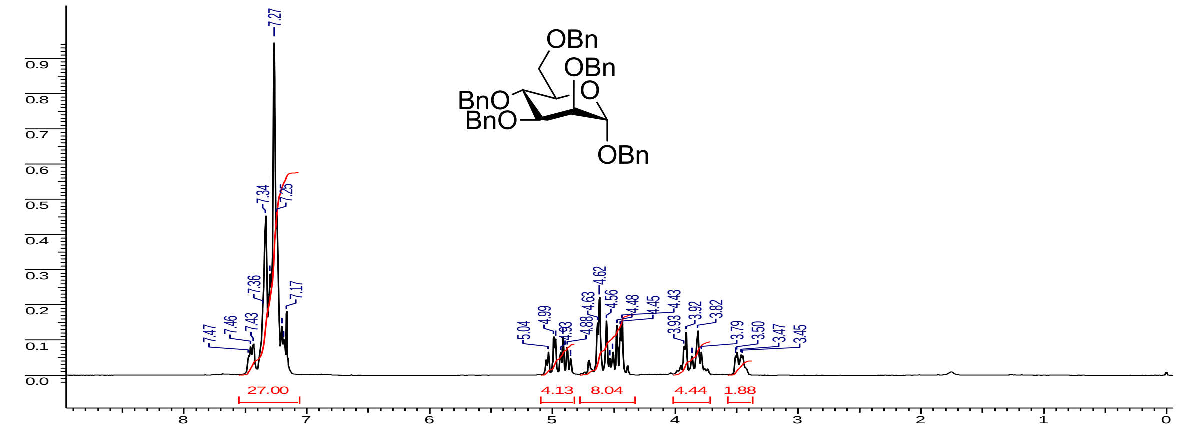

^{13}C NMR Spectrum (50.32 MHz, $CDCl_3$) of Compound **6**

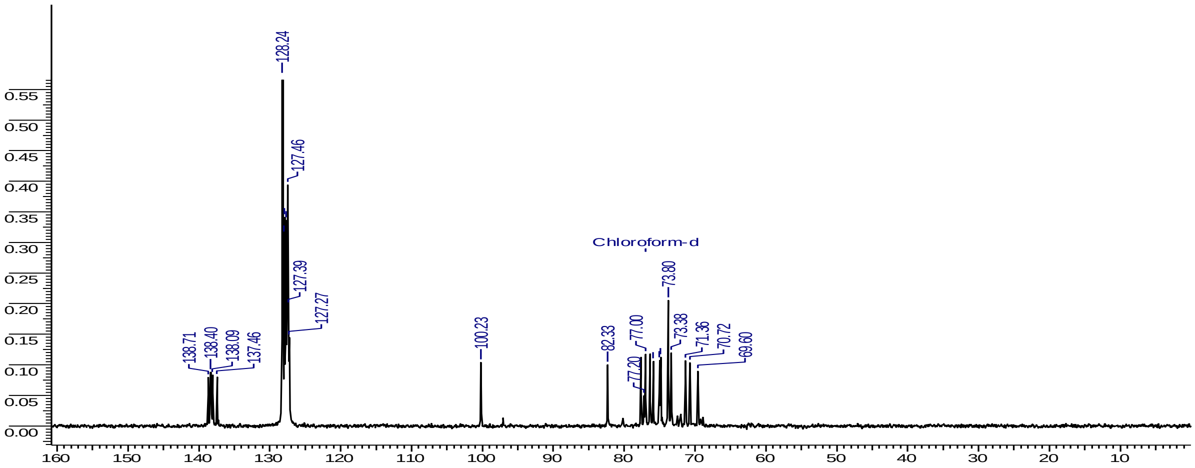

DEPT NMR Spectrum (50.32 MHz, $CDCl_3$) of Compound **6**

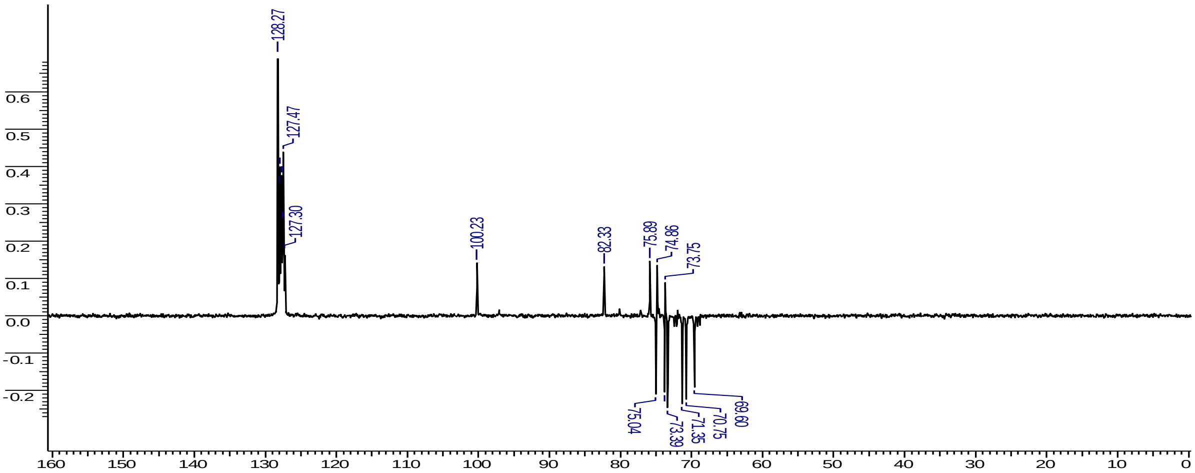

^{1}H NMR Spectrum (200.13 MHz, $CDCl_3$) of Compound **7**

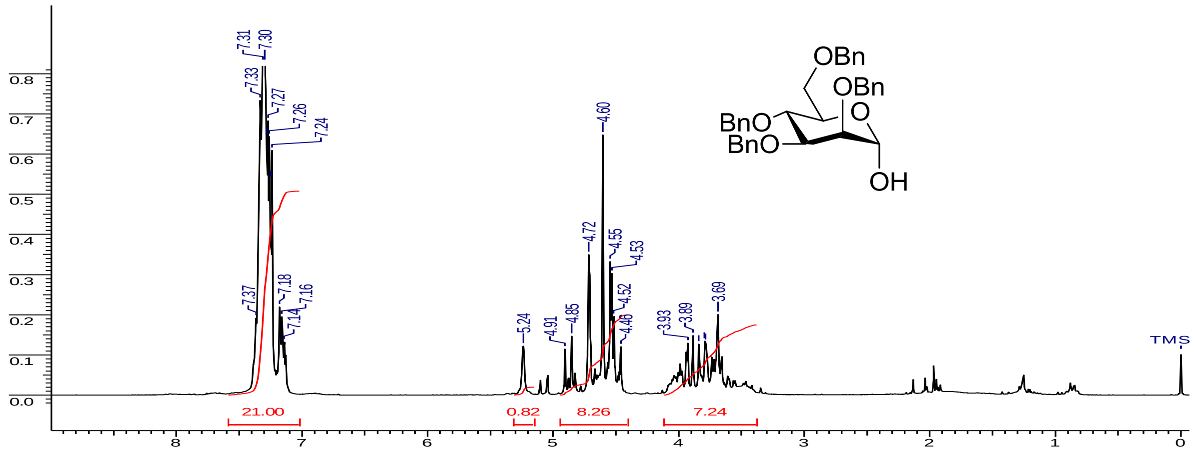

^{13}C NMR Spectrum (50.32 MHz, $CDCl_3$) of Compound **7**

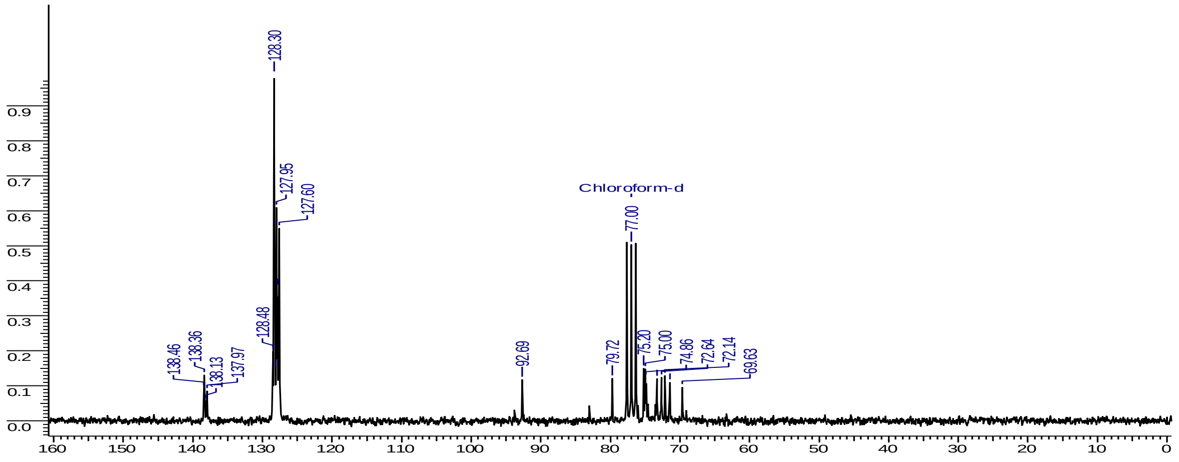

DEPT NMR Spectrum (50.32 MHz, $CDCl_3$) of Compound **7**

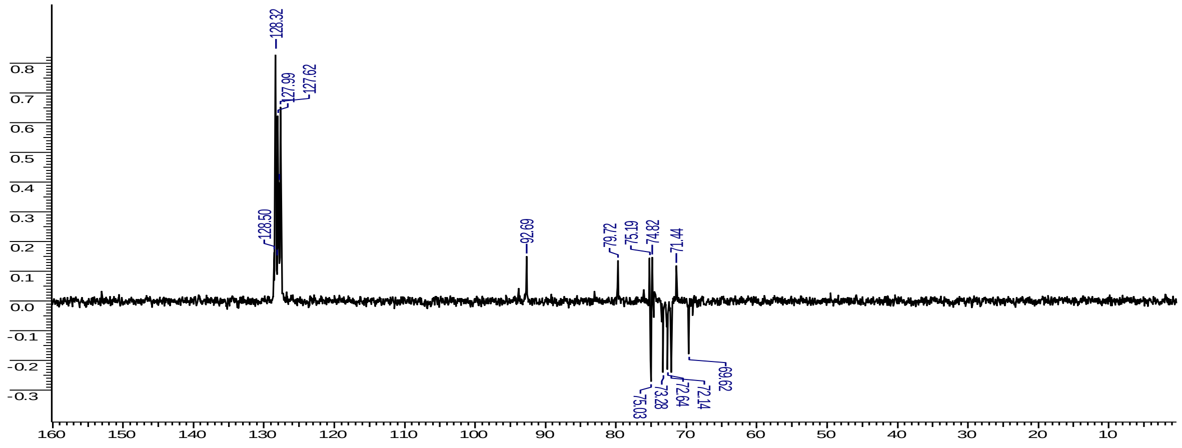

^{1}H NMR Spectrum (399.78 MHz, $CDCl_3$) of Compound **8**

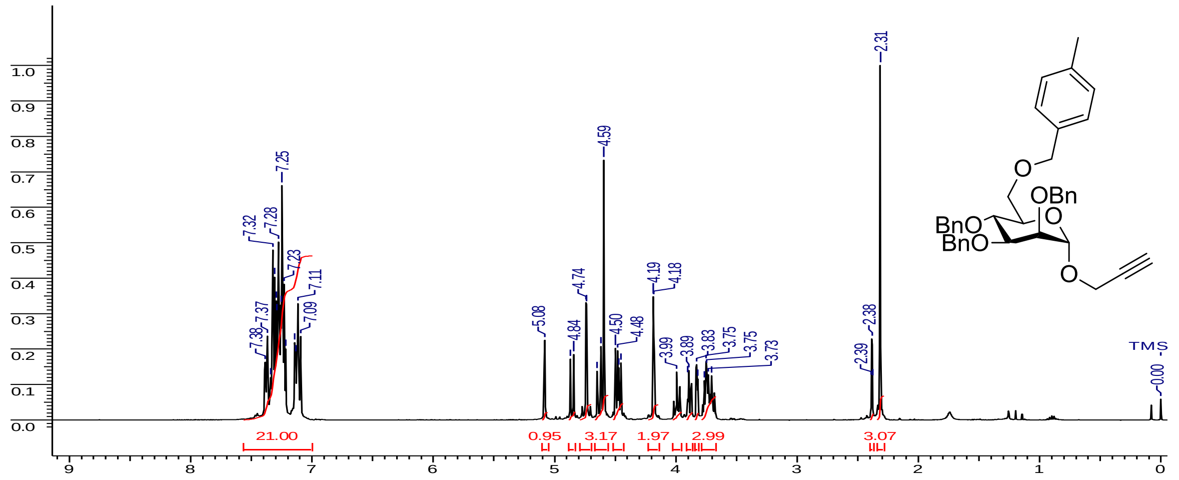

^{13}C NMR Spectrum (100.53 MHz, $CDCl_3$) of Compound **8**

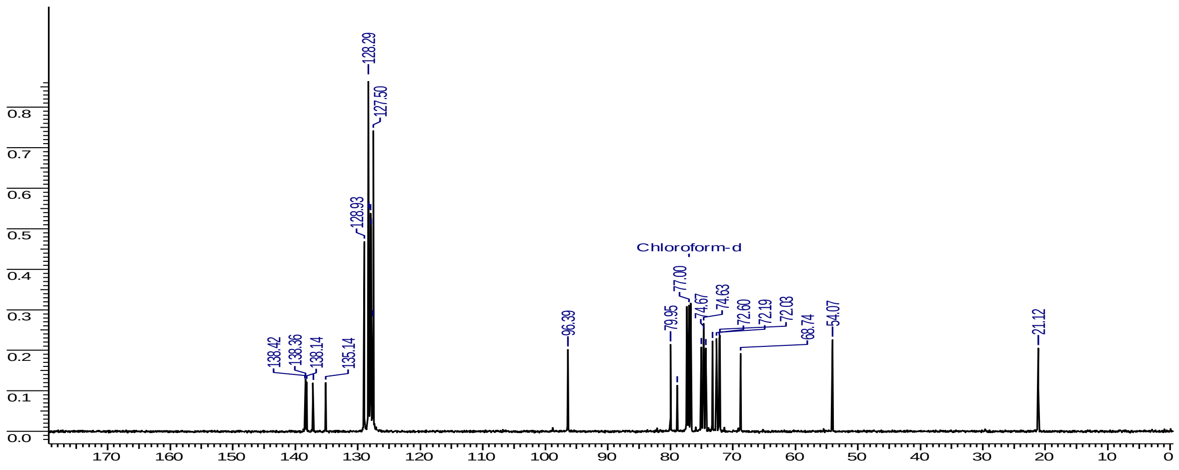

DEPT NMR Spectrum (100.53 MHz, $CDCl_3$) of Compound **8**

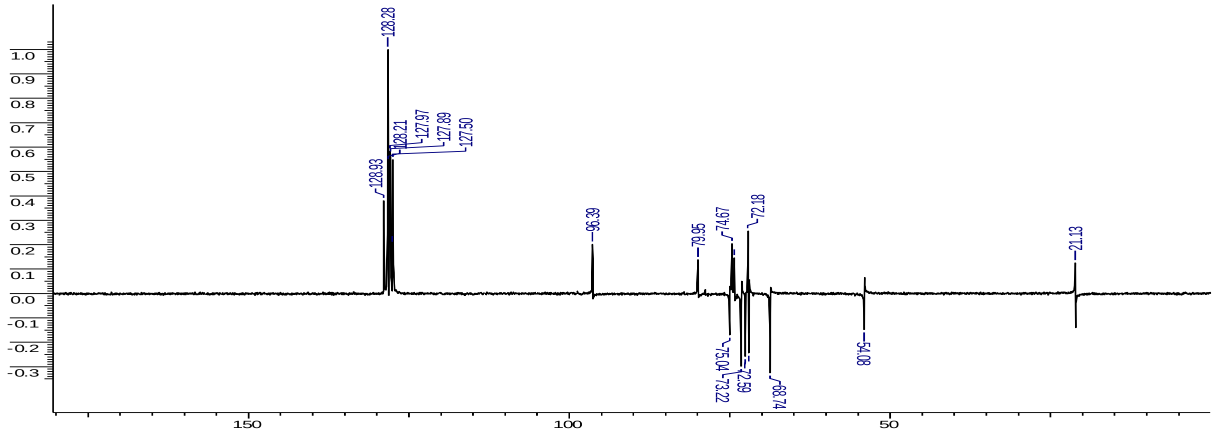

^{1}H NMR Spectrum (399.78 MHz, $CDCl_3$) of Compound **12**

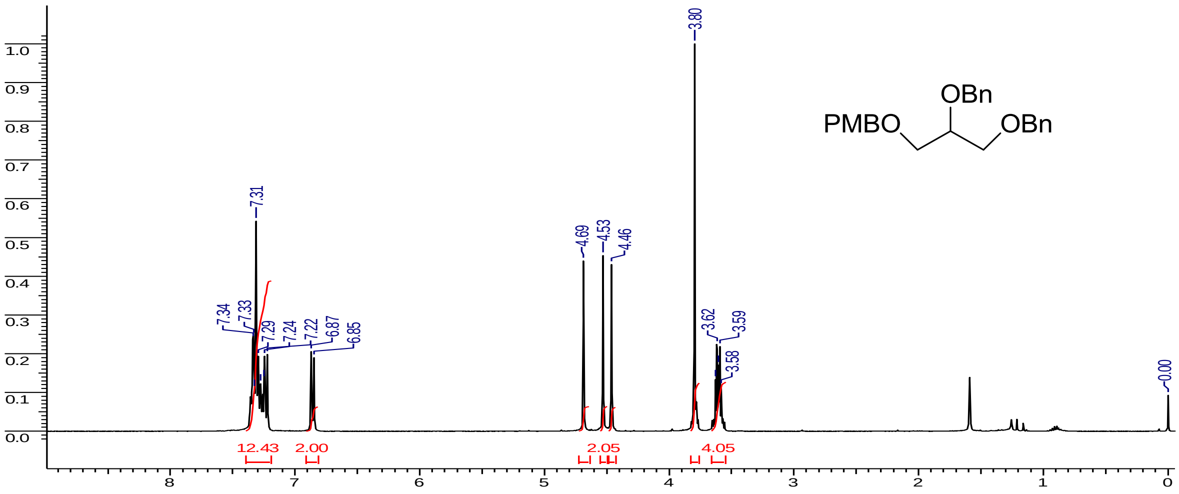

^{13}C NMR Spectrum (100.53 MHz, $CDCl_3$) of Compound **12**

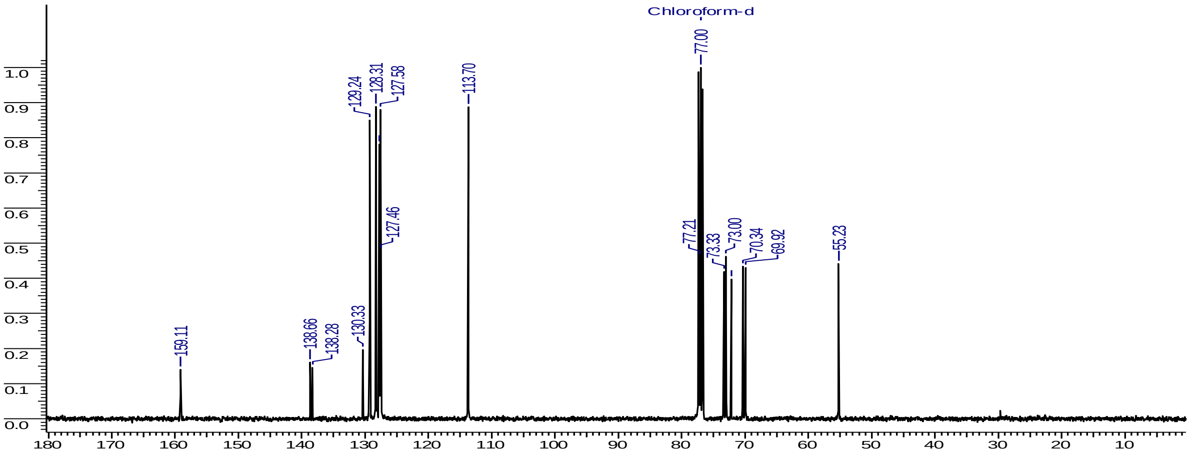

DEPT NMR Spectrum (100.53 MHz, $CDCl_3$) of Compound **12**

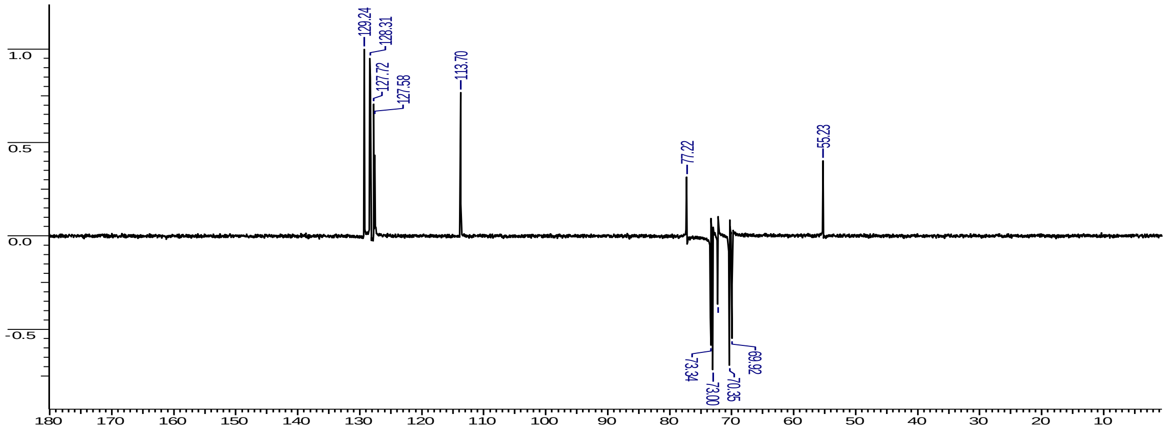

^{1}H NMR Spectrum (200.13 MHz, $CDCl_3$) of Compound **13**

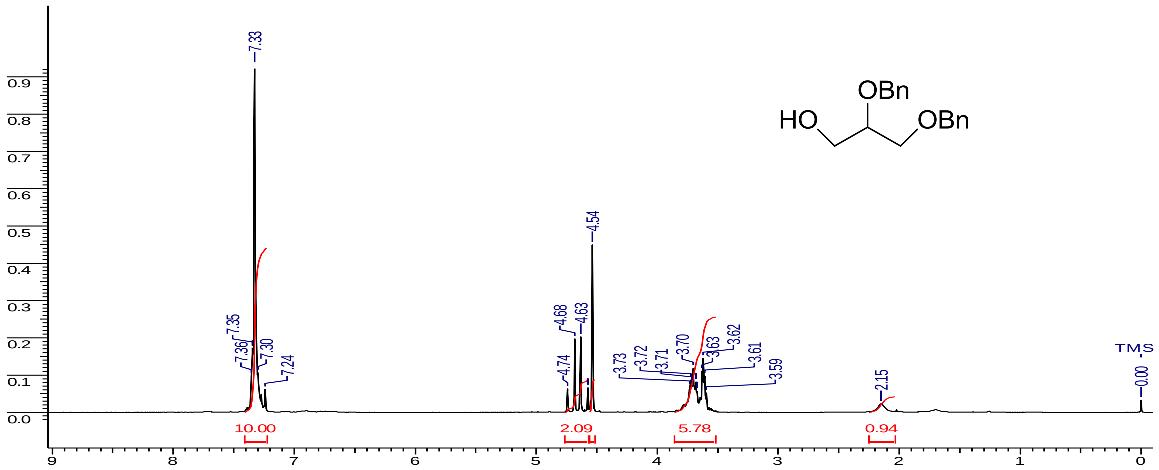

^{13}C NMR Spectrum (50.32 MHz, $CDCl_3$) of Compound **13**

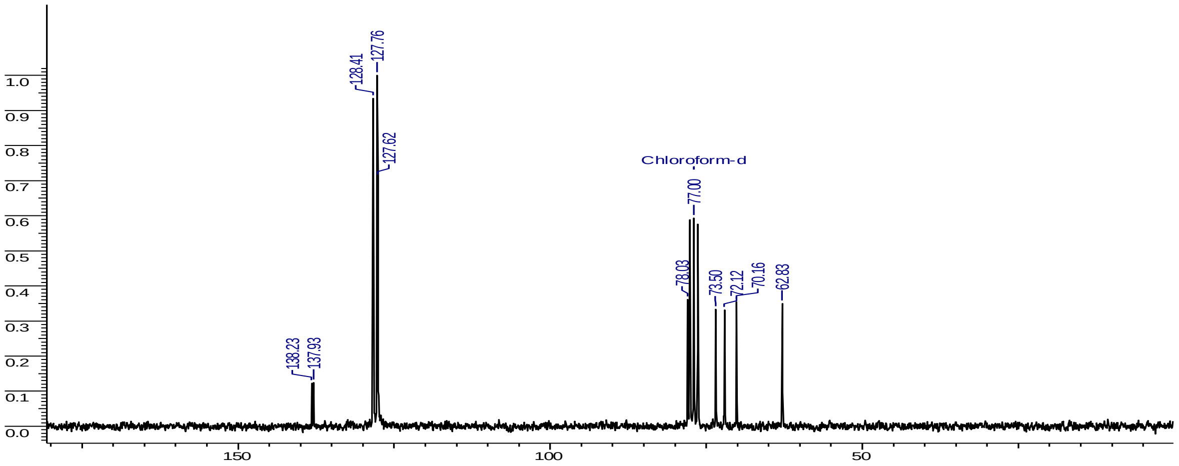

DEPT NMR Spectrum (50.32 MHz, $CDCl_3$) of Compound **13**

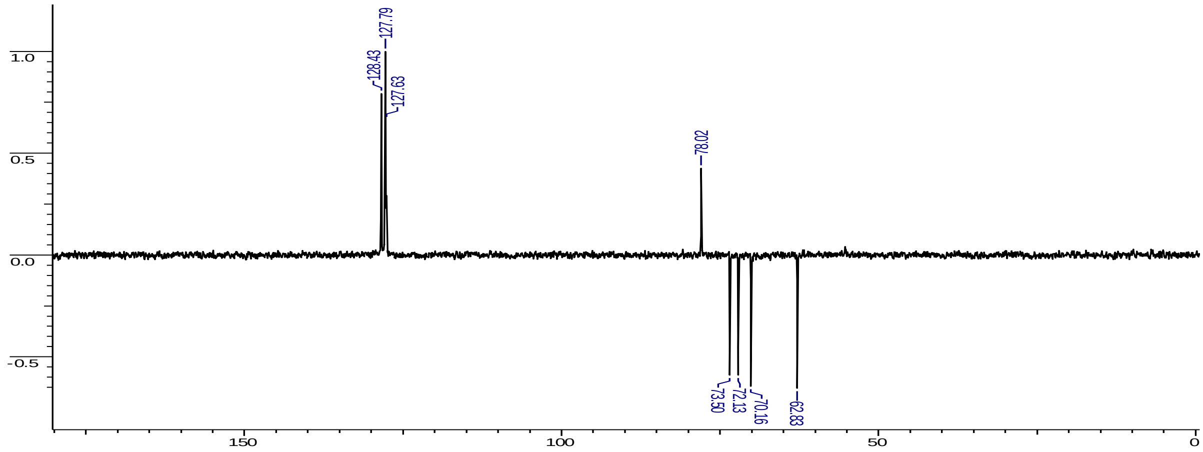

^{1}H NMR Spectrum (399.78 MHz, $CDCl_3$) of Compound **14**

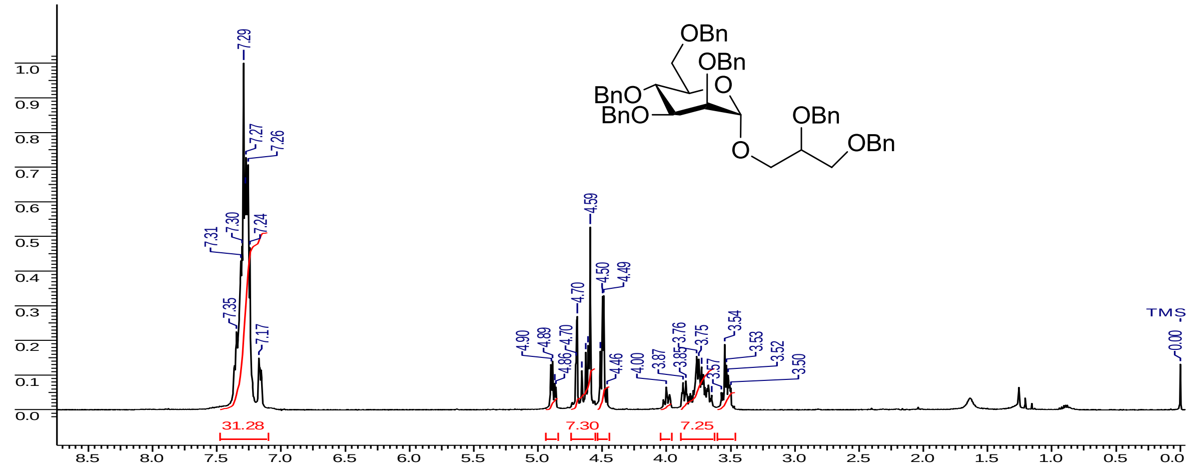

^{13}C NMR Spectrum (100.53 MHz, $CDCl_3$) of Compound **14**

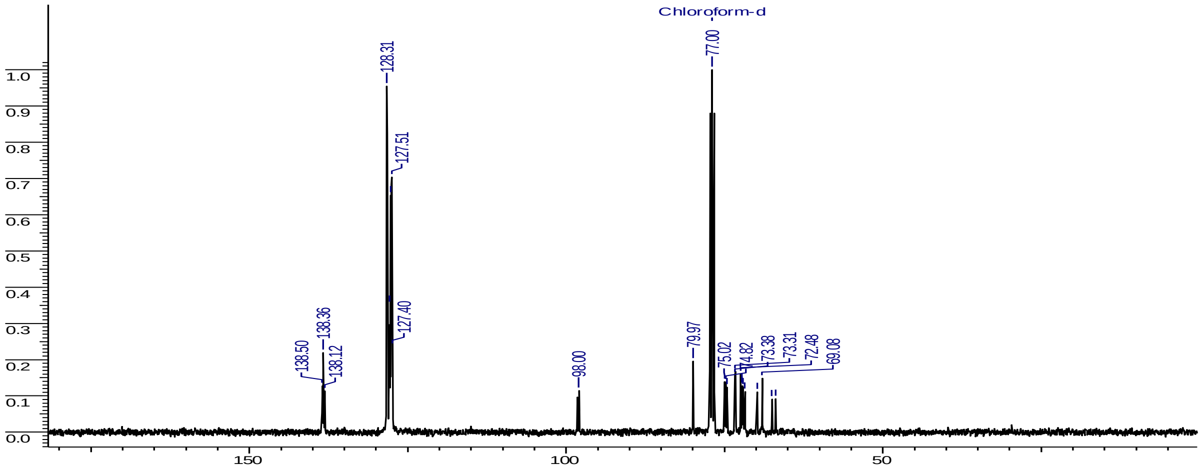

DEPT NMR Spectrum (100.53 MHz, $CDCl_3$) of Compound **14**

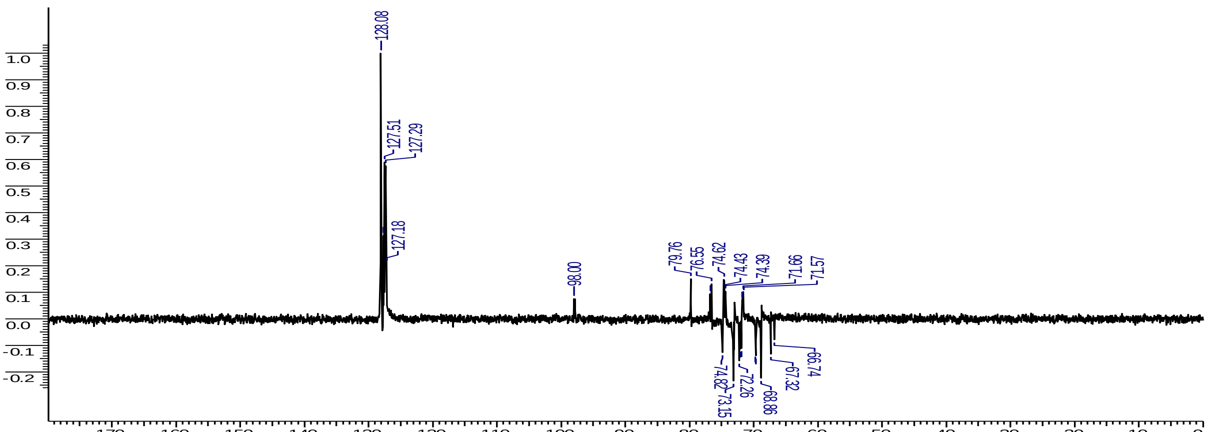

^{1}H NMR Spectrum (200.13 MHz, $CDCl_3$) of Compound **15j**

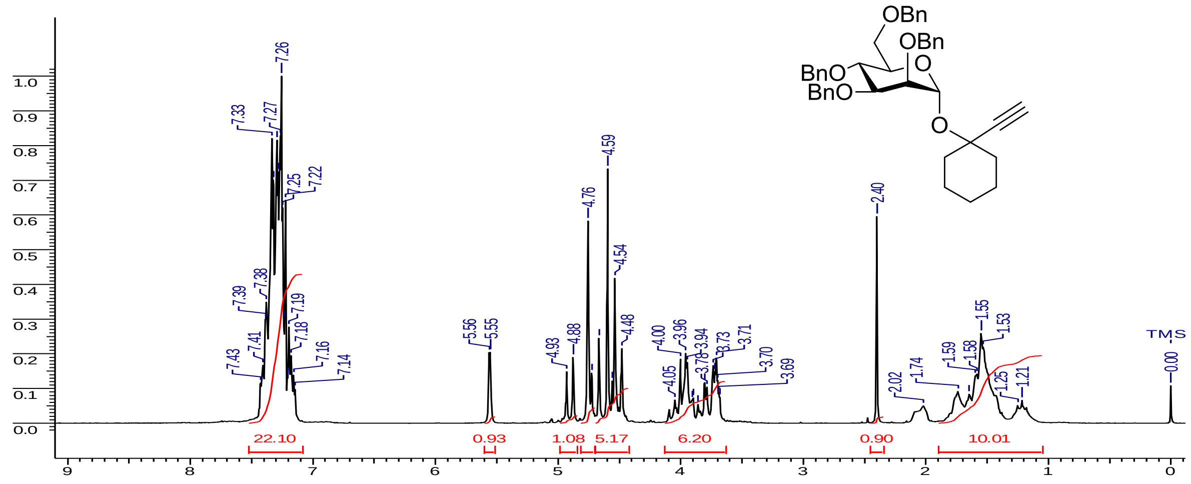

^{13}C NMR Spectrum (50.32 MHz, $CDCl_3$) of Compound **15j**

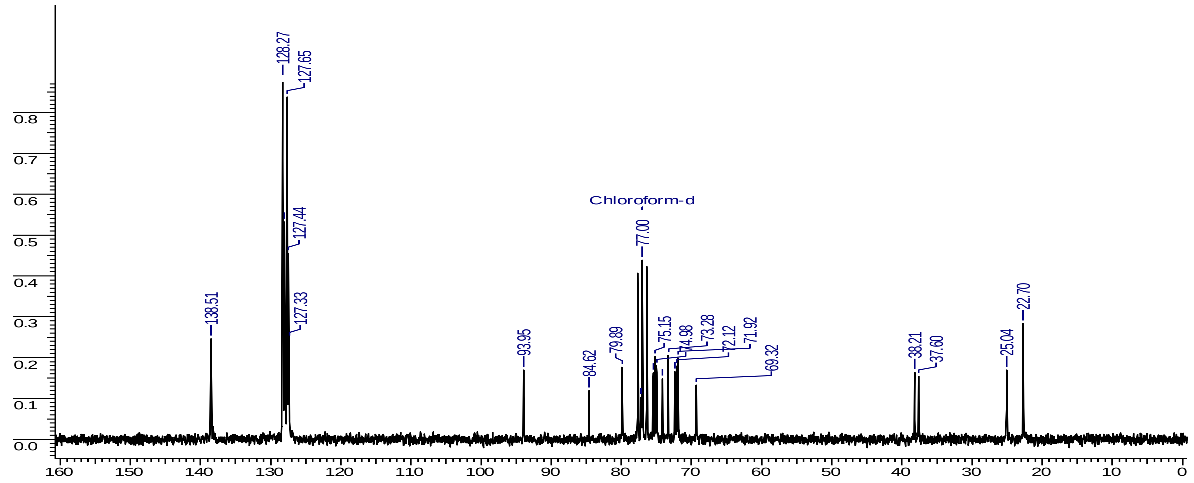

DEPT NMR Spectrum (50.32 MHz, $CDCl_3$) of Compound **15j**

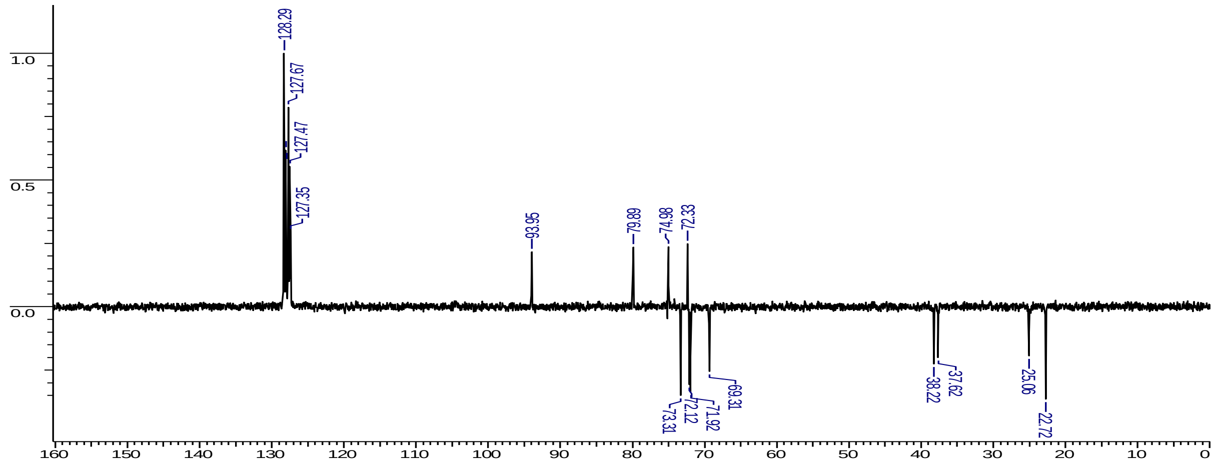

^{1}H NMR Spectrum (200.13 MHz, $CDCl_3$) of Compound **15k**

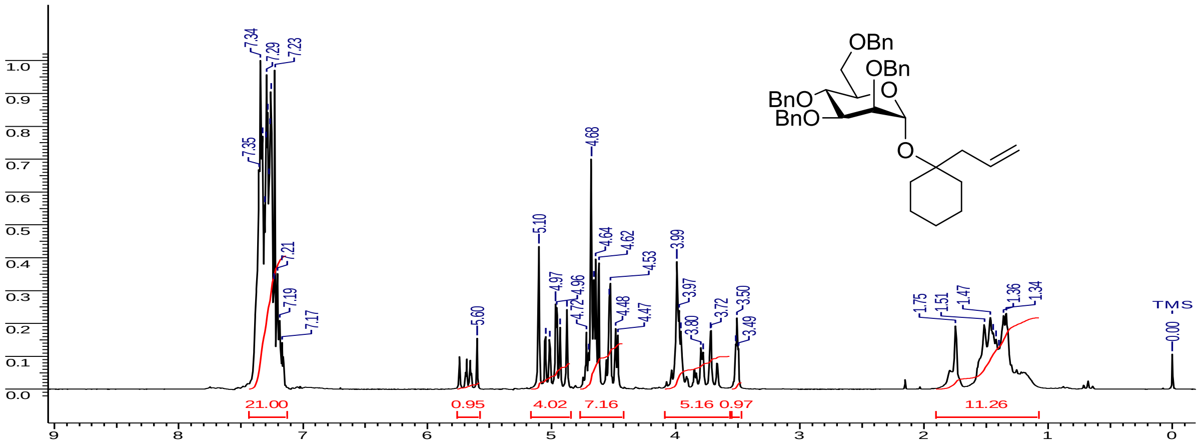

^{13}C NMR Spectrum (50.32 MHz, $CDCl_3$) of Compound **15k**

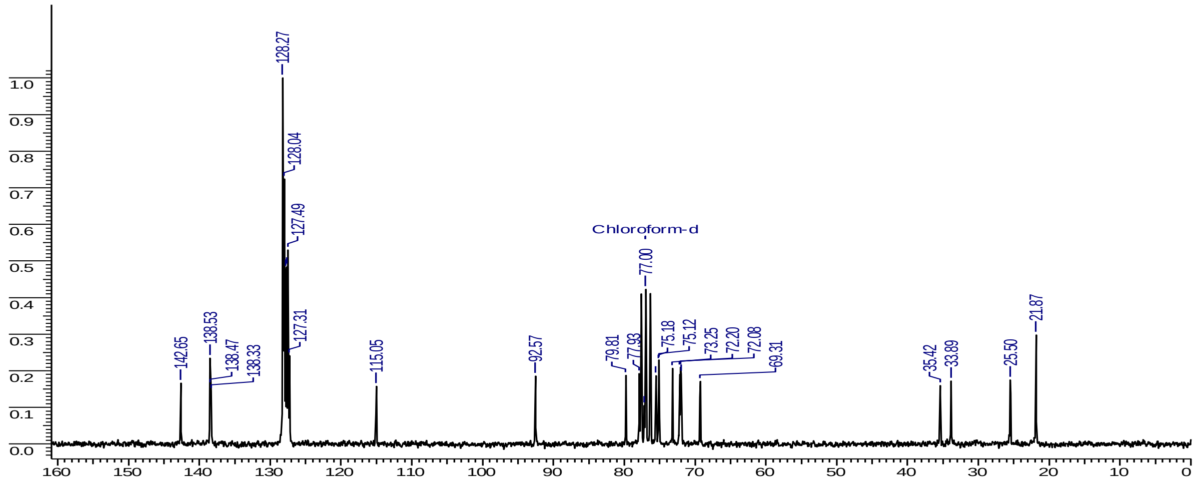

DEPT NMR Spectrum (50.32 MHz, $CDCl_3$) of Compound **15k**

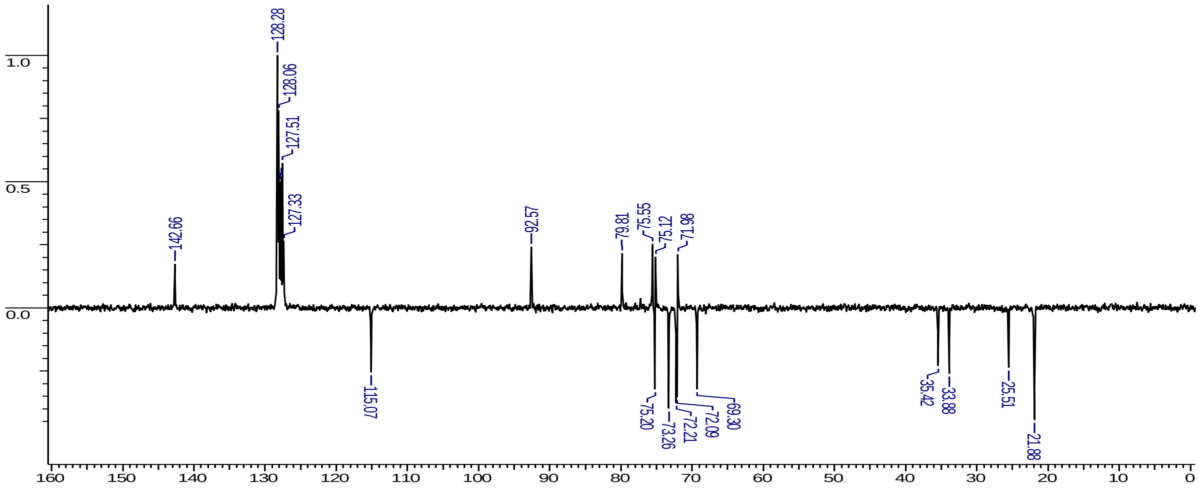

^{1}H NMR Spectrum (200.13 MHz, $CDCl_3$) of Compound **15l**

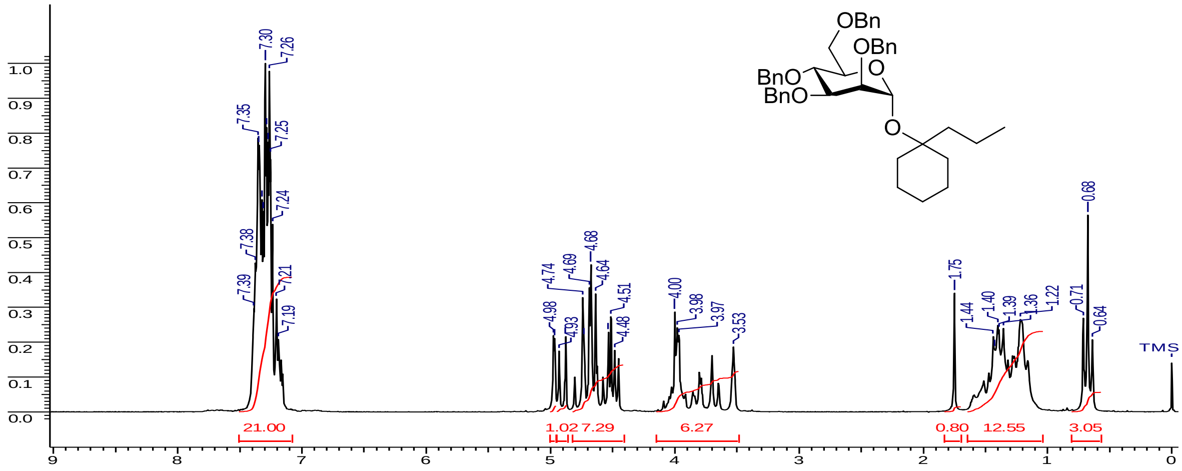

^{13}C NMR Spectrum (50.32 MHz, $CDCl_3$) of Compound **15l**

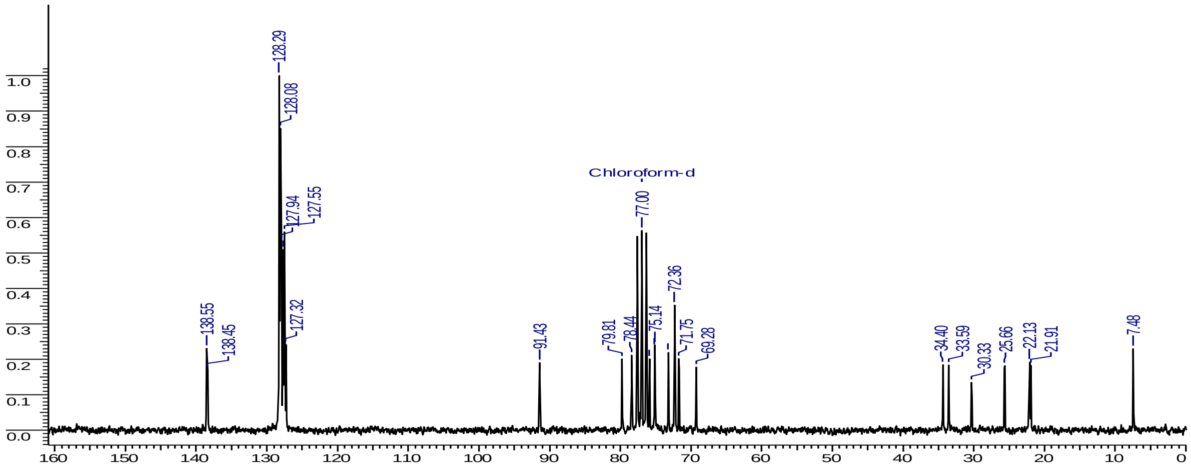

DEPT NMR Spectrum (50.32 MHz, $CDCl_3$) of Compound **15l**

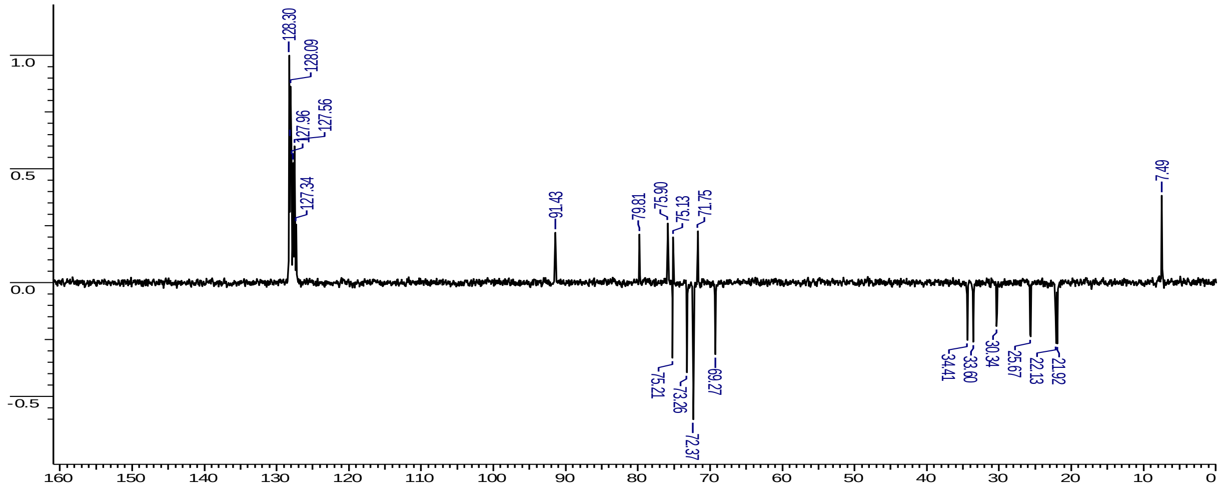

^{1}H NMR Spectrum (200.13 MHz, $CDCl_3$) of Compound **17**

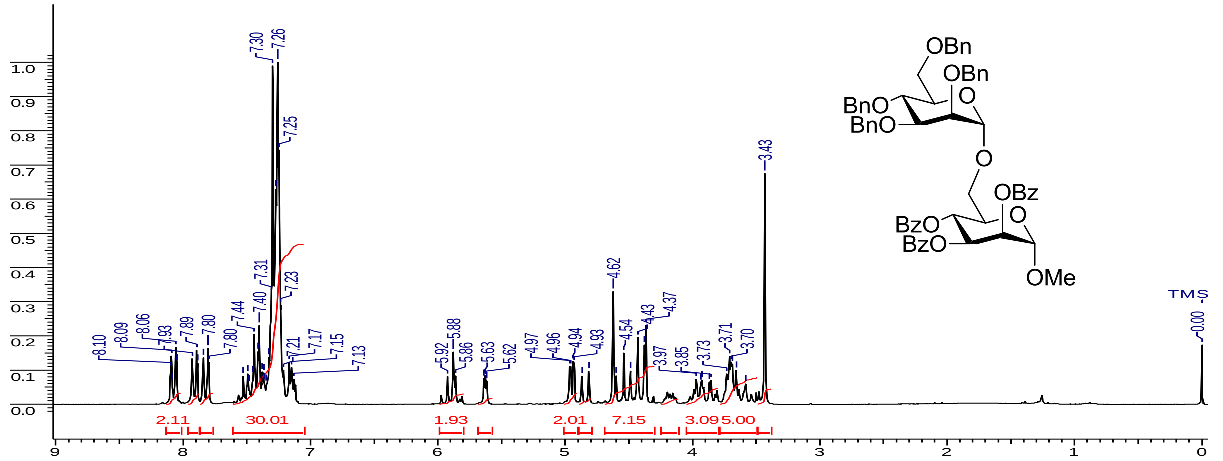

^{13}C NMR Spectrum (125.76 MHz, $CDCl_3$) of Compound **17**

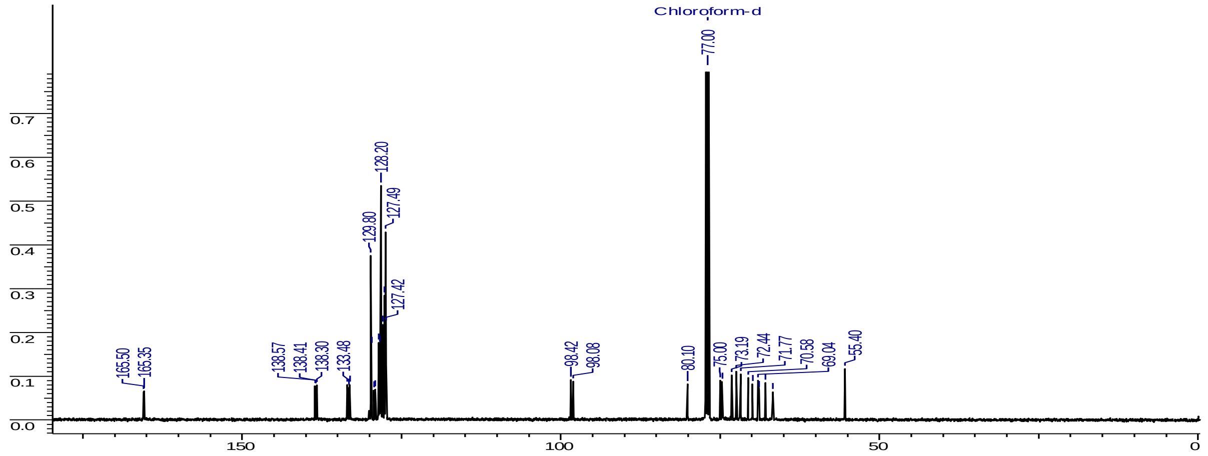

DEPT NMR Spectrum (125.76 MHz, $CDCl_3$) of Compound **17**

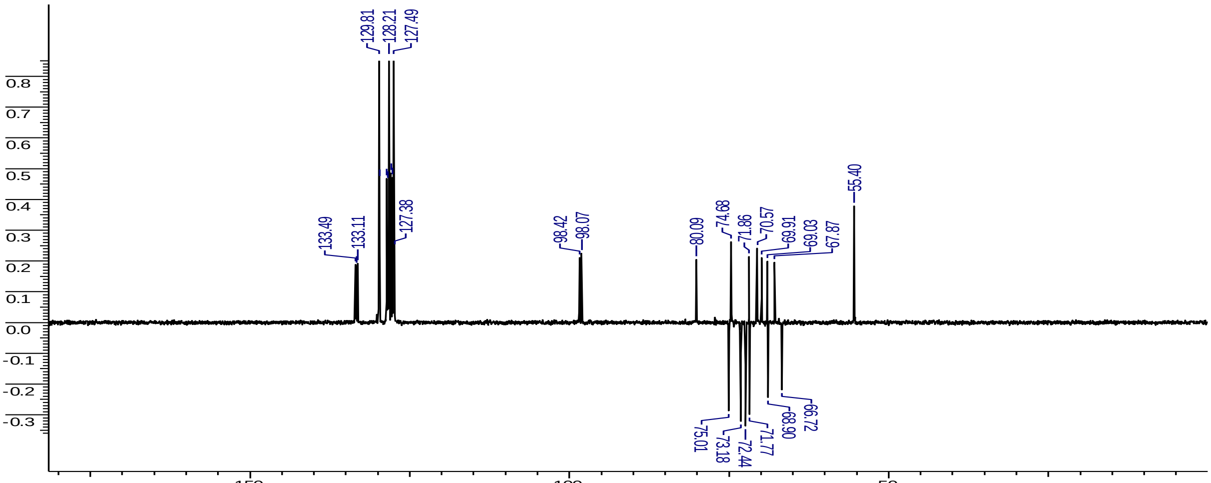

^{1}H NMR Spectrum (200.13 MHz, $CDCl_3$) of Compound **24**

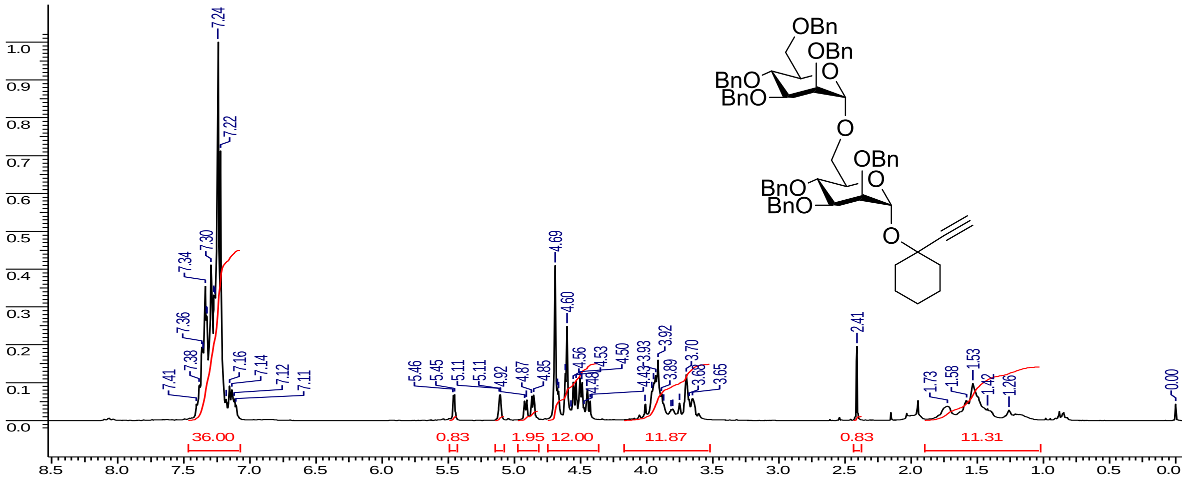

^{13}C NMR Spectrum (50.32 MHz, $CDCl_3$) of Compound **24**

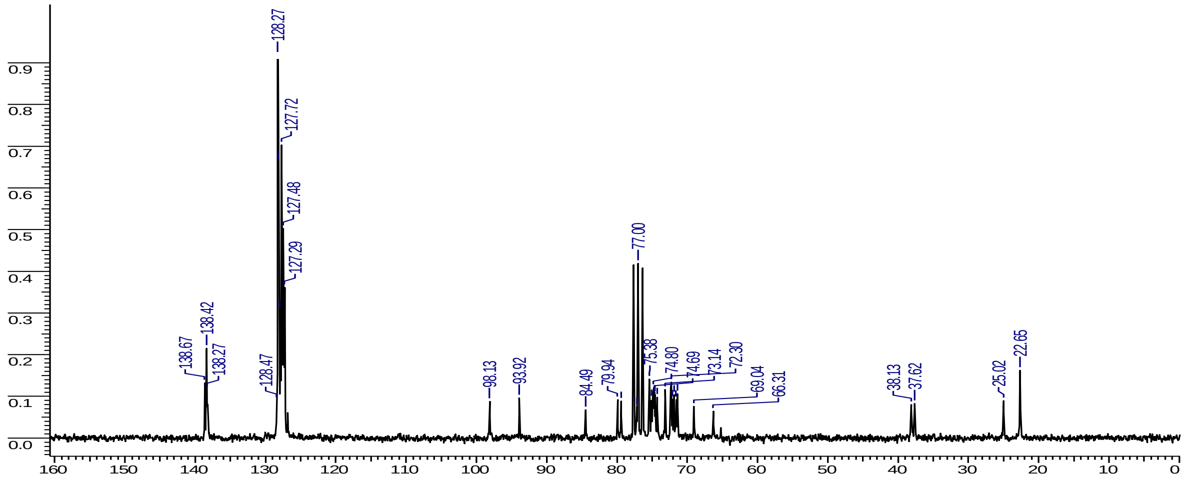

DEPT NMR Spectrum (50.32 MHz, $CDCl_3$) of Compound **24**

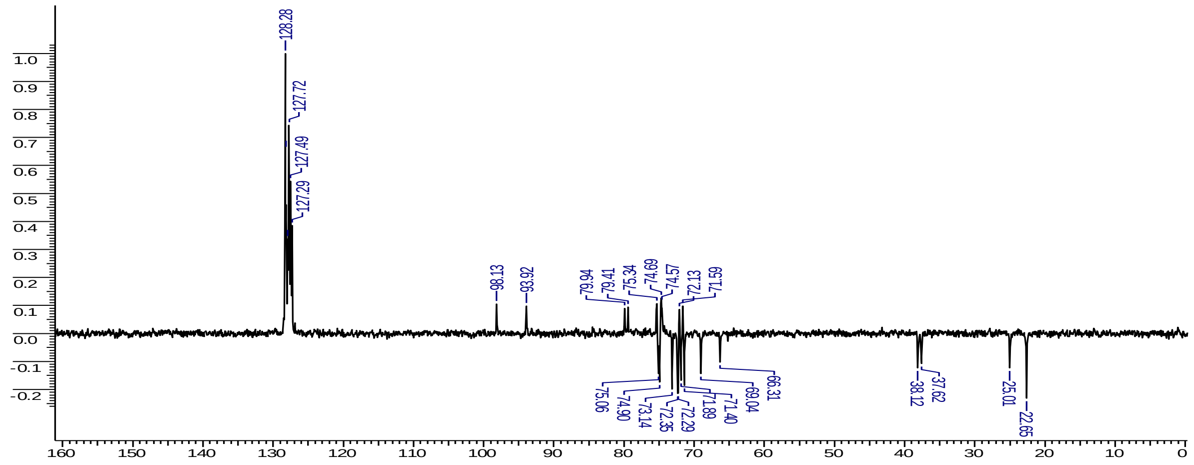

^{1}H NMR Spectrum (500.13 MHz, $CDCl_3$) of Compound **25**

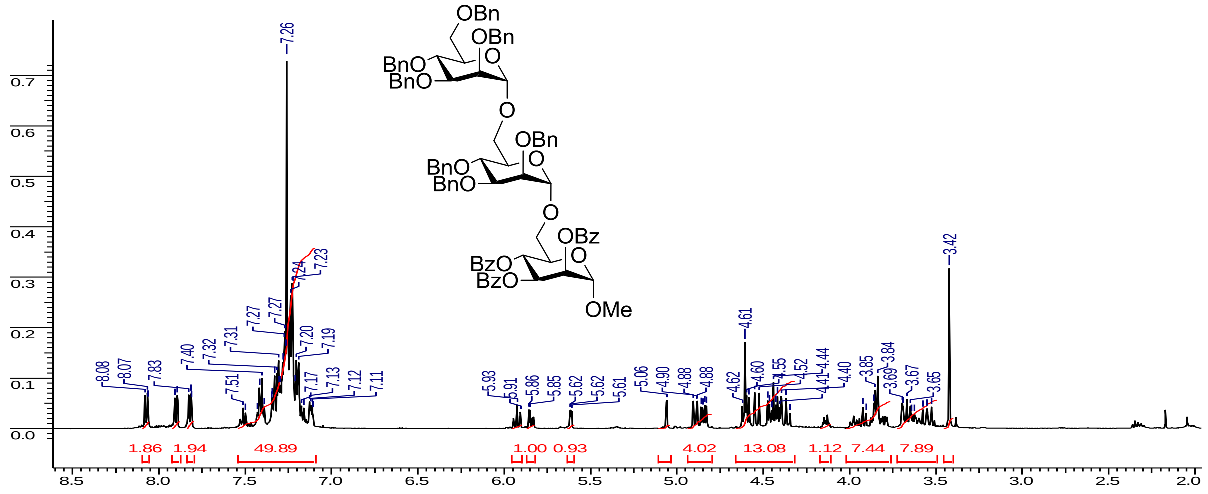

^{13}C NMR Spectrum (125.76 MHz, $CDCl_3$) of Compound **25**

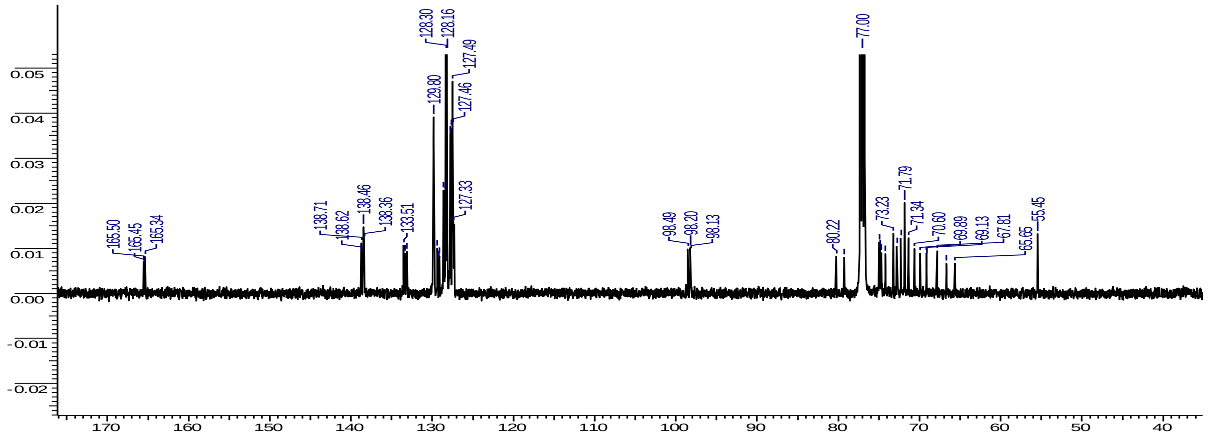

DEPT NMR Spectrum (125.76 MHz, $CDCl_3$) of Compound **25**

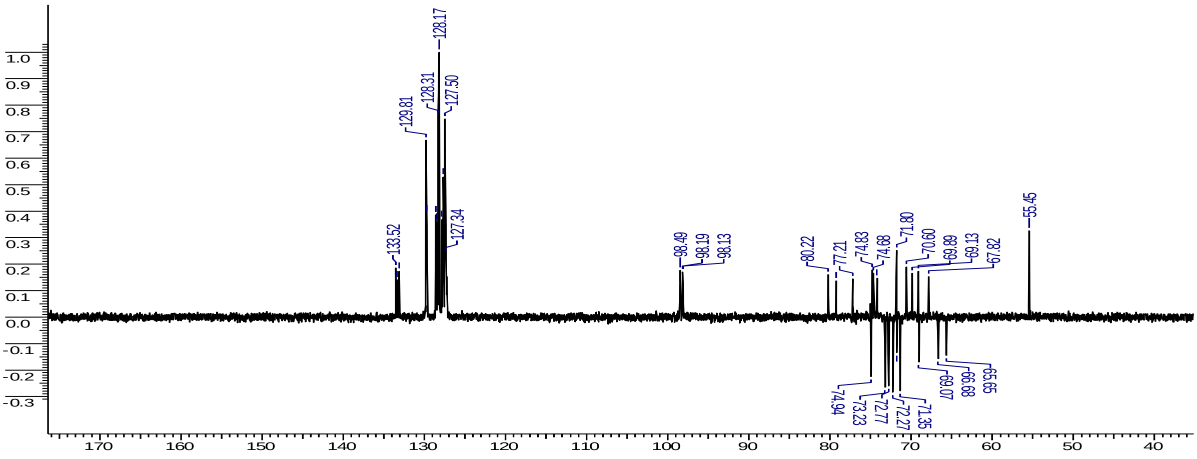

^{1}H NMR Spectrum (399.78 MHz, $CDCl_3$) of Compound **26**

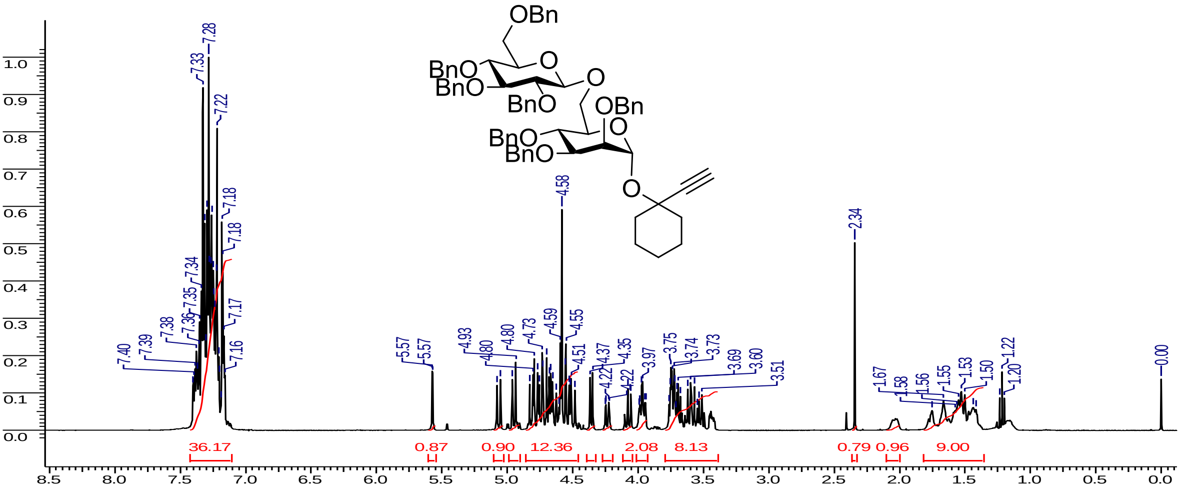

^{13}C NMR Spectrum (100.53 MHz, $CDCl_3$) of Compound **26**

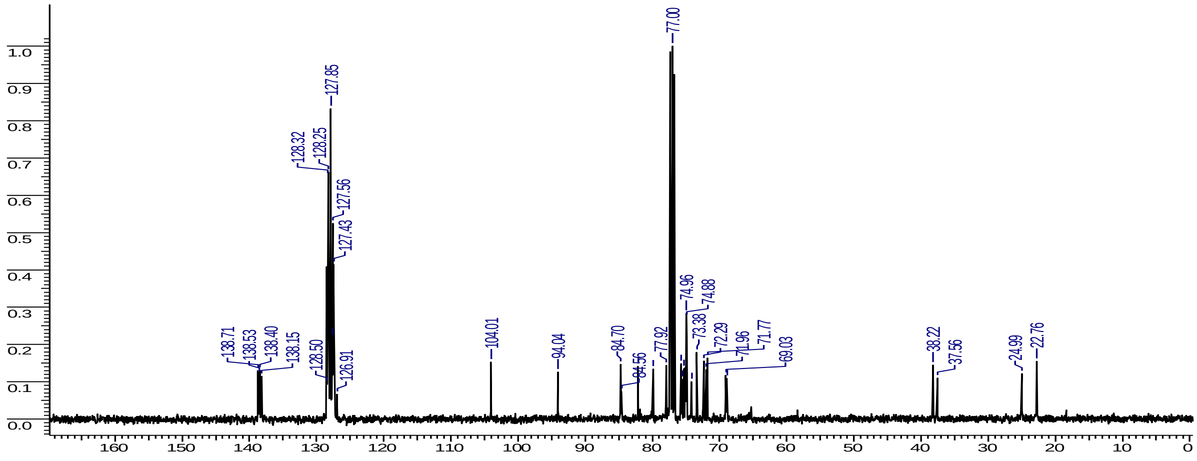

DEPT NMR Spectrum (100.53 MHz, $CDCl_3$) of Compound **26**

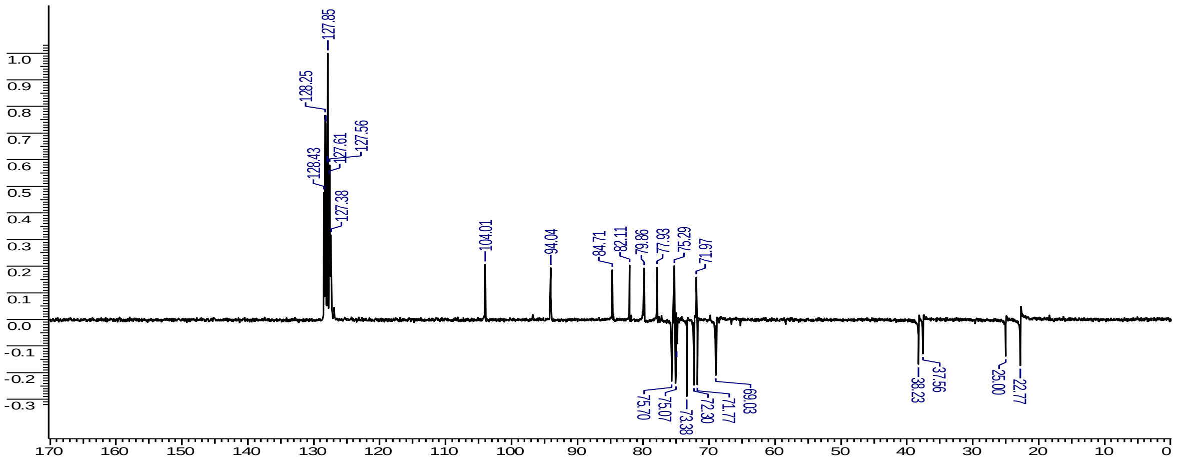

^{1}H NMR Spectrum (399.78 MHz, $CDCl_3$) of Compound **27**

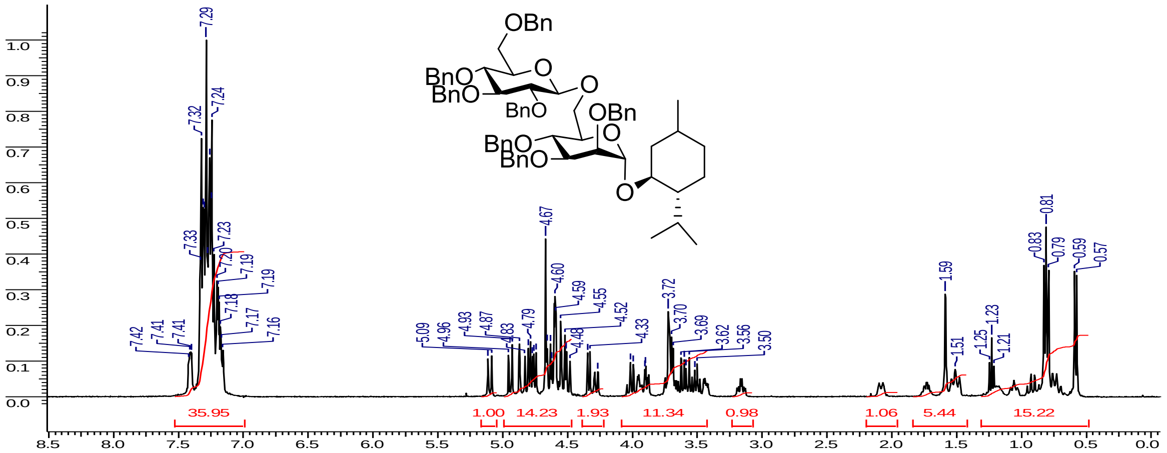

^{13}C NMR Spectrum (100.53 MHz, $CDCl_3$) of Compound **27**

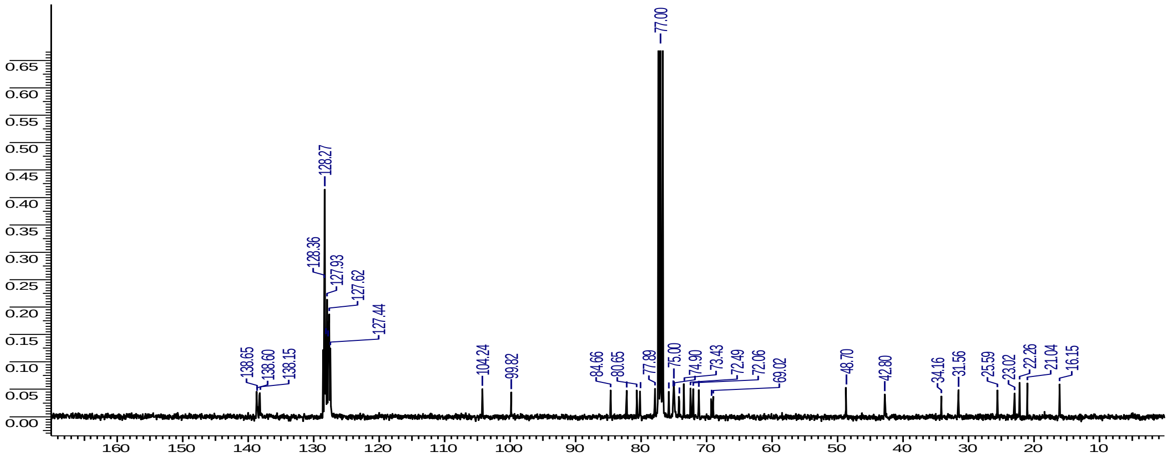

DEPT NMR Spectrum (100.53 MHz, $CDCl_3$) of Compound **27**

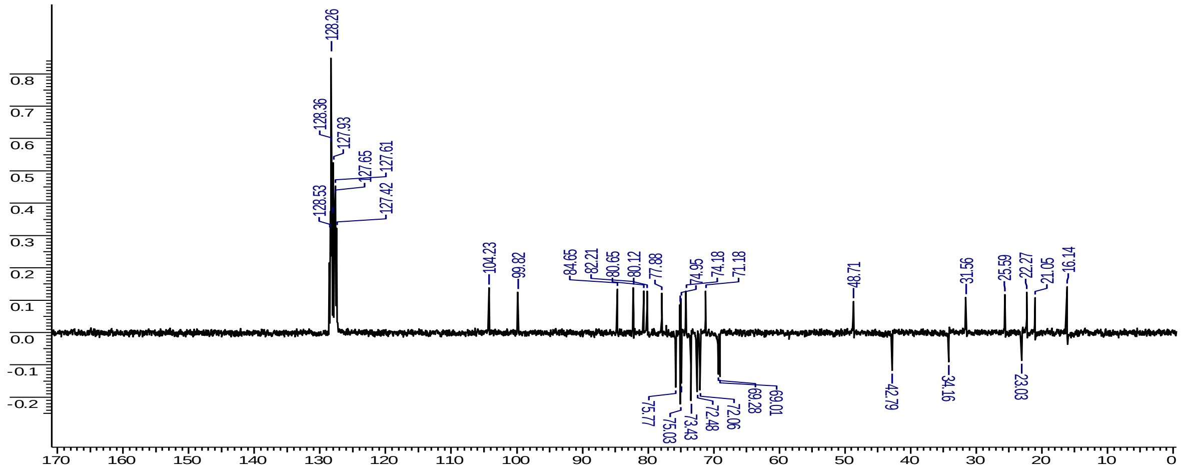

^{1}H NMR Spectrum (399.78 MHz, $CDCl_3$) of Compound **29**

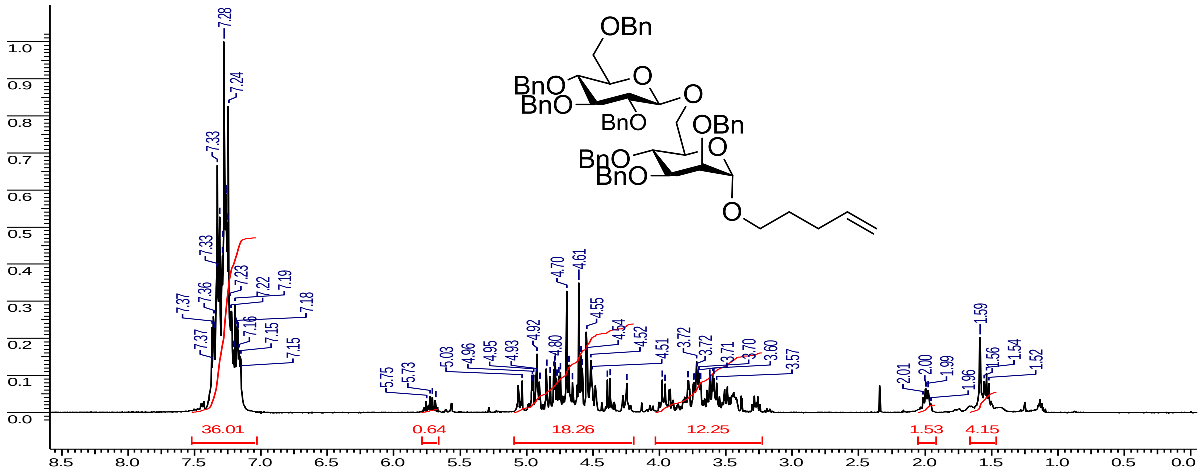

^{13}C NMR Spectrum (100.53 MHz, $CDCl_3$) of Compound **29**

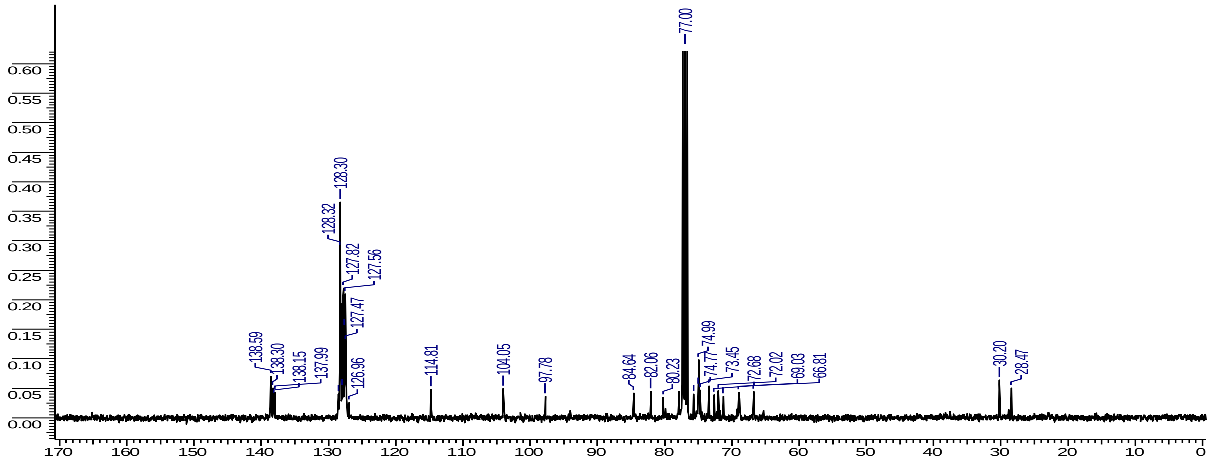

DEPT NMR Spectrum (100.53 MHz, $CDCl_3$) of Compound **29**

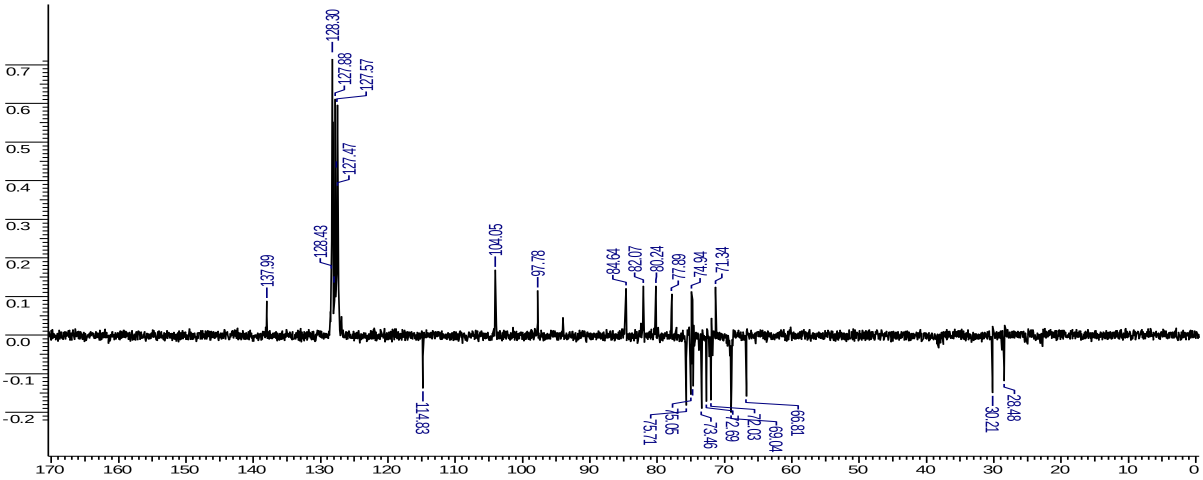

^{1}H NMR Spectrum (399.78 MHz, $CDCl_3$) of Compound **31**

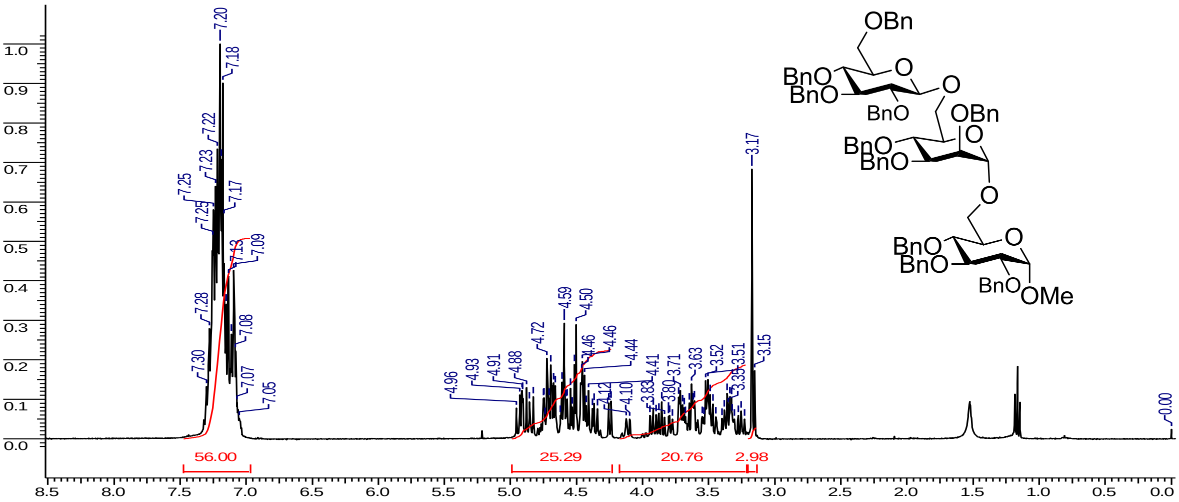

^{13}C NMR Spectrum (100.53 MHz, $CDCl_3$) of Compound **31**

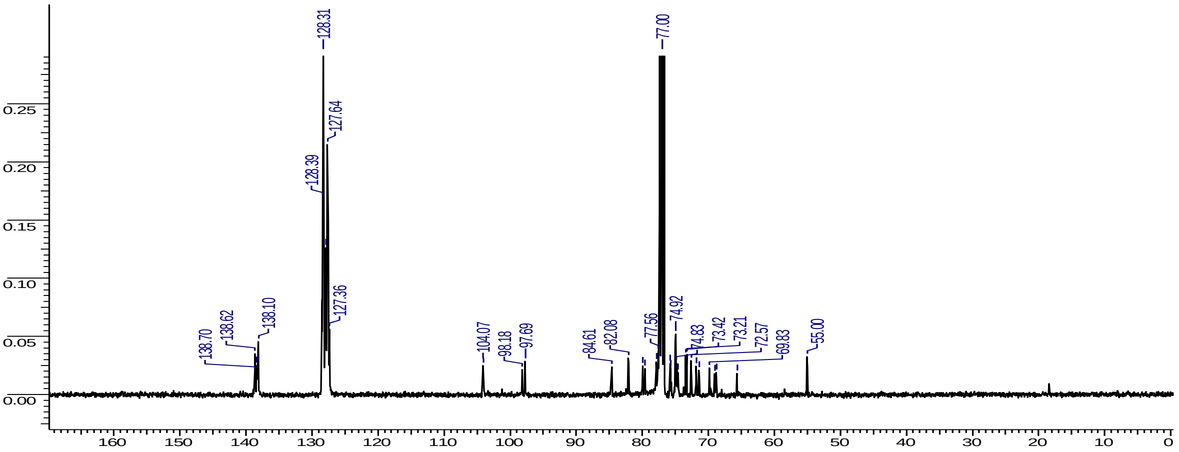

DEPT NMR Spectrum (100.53 MHz, $CDCl_3$) of Compound **31**

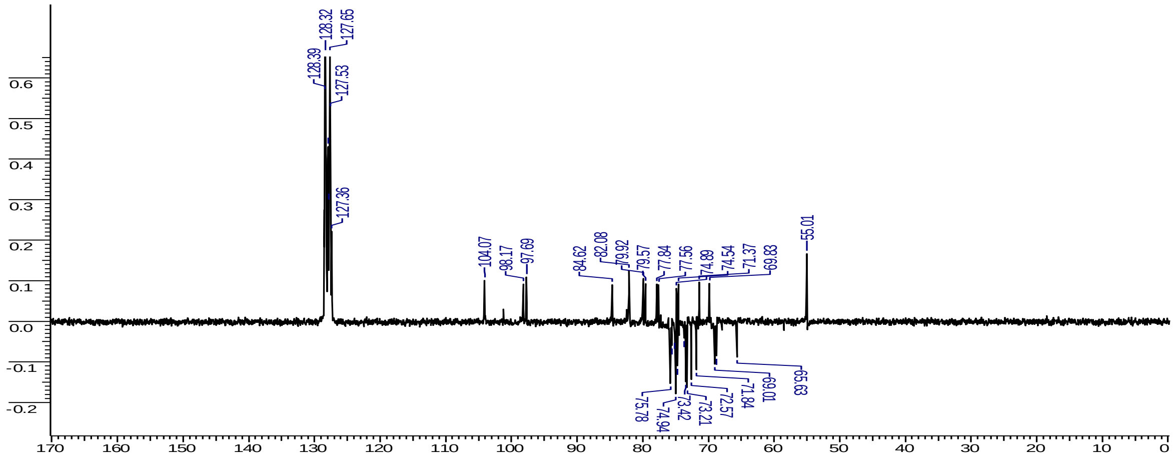

^{1}H NMR Spectrum (399.78 MHz, $CDCl_3$) of Compound **32**

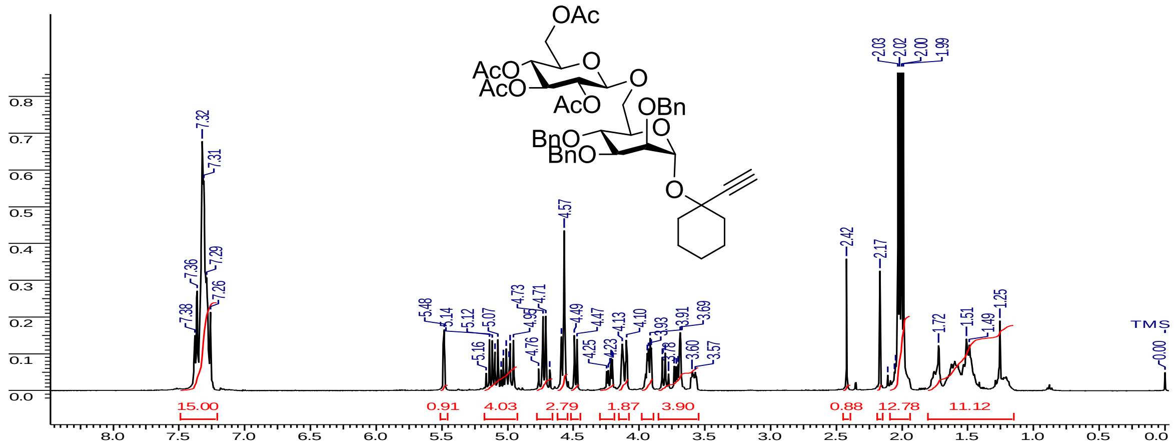

^{13}C NMR Spectrum (100.53 MHz, $CDCl_3$) of Compound **32**

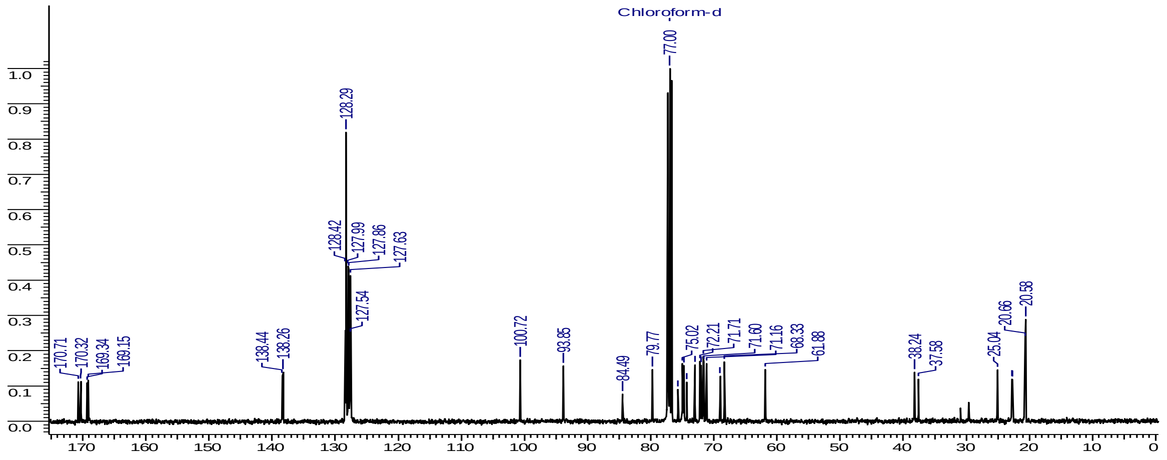

DEPT NMR Spectrum (100.53 MHz, $CDCl_3$) of Compound **32**

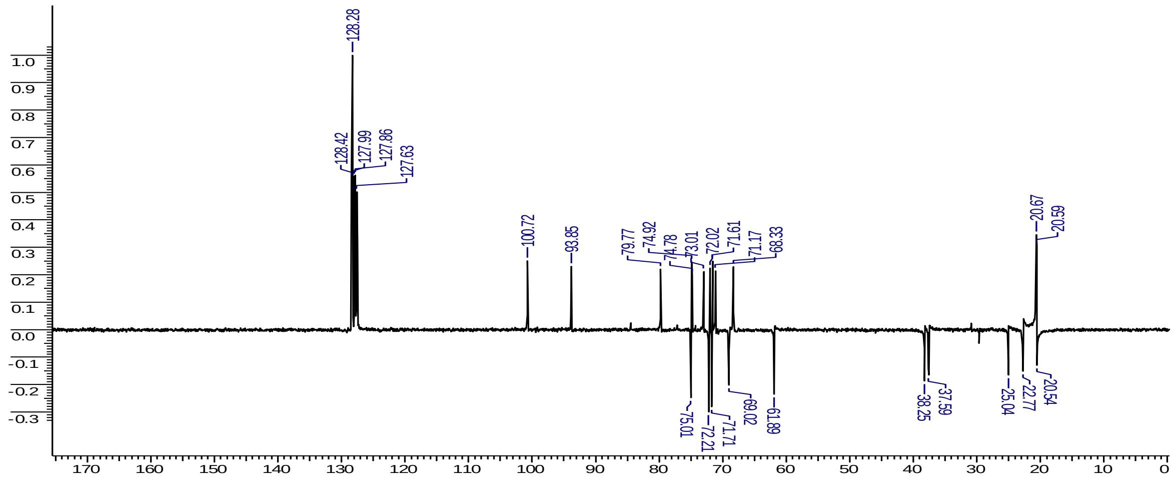

[1] H NMR Spectrum (399.78 MHz, $CDCl_3$) of Compound **33**

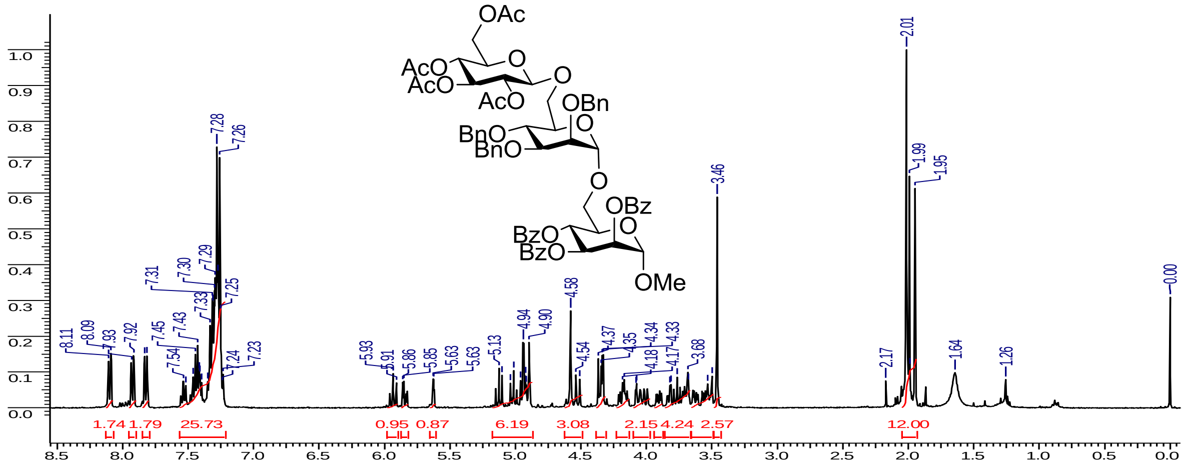

[13] C NMR Spectrum (100.53 MHz, $CDCl_3$) of Compound **33**

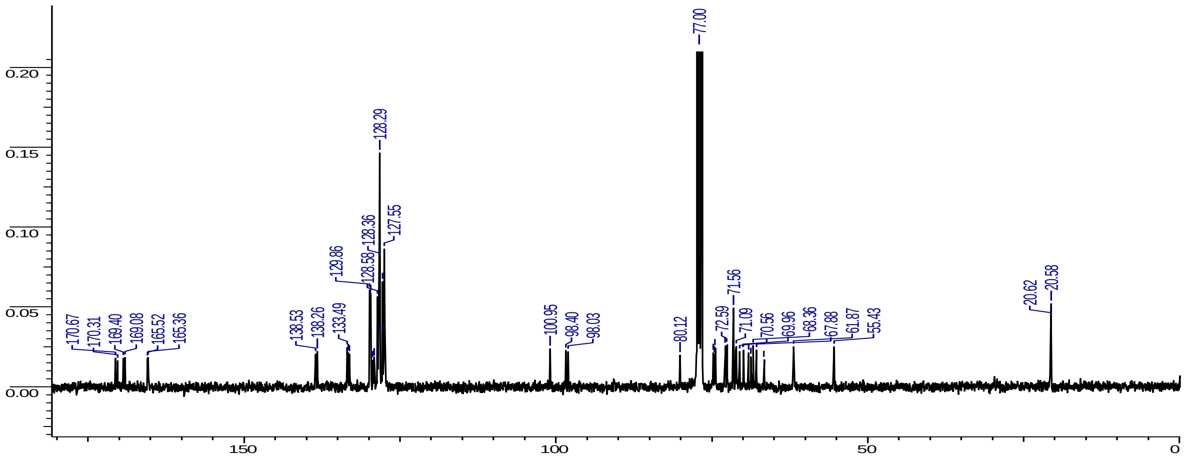

DEPT NMR Spectrum (100.53 MHz, $CDCl_3$) of Compound **33**

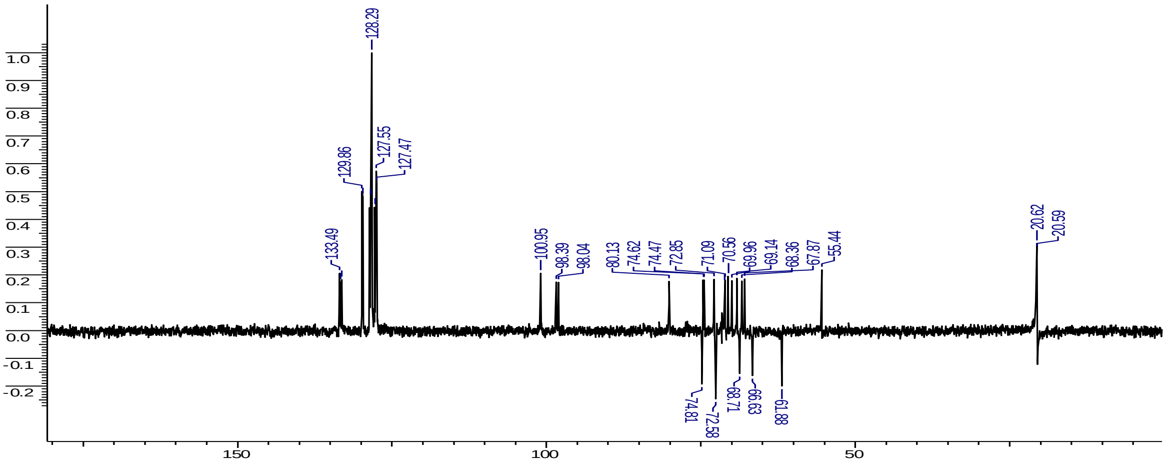

References

1. Kayastha, A.K.; Hotha, S. *Tetrahedron Lett.* **2010,** *51*, 5269–5272.

2. Kayastha, A. K.; Hotha, S. *Chem. Commun.* **2012,** *48*, 7161–7163.
